# A network pharmacology approach reveals new candidate caloric restriction mimetics in *C. elegans*

**DOI:** 10.1111/acel.12432

**Published:** 2015-12-16

**Authors:** Shaun Calvert, Robi Tacutu, Samim Sharifi, Rute Teixeira, Pratul Ghosh, João Pedro de Magalhães

**Affiliations:** ^1^ Integrative Genomics of Ageing GroupInstitute of Integrative BiologyUniversity of LiverpoolLiverpoolUK

**Keywords:** aging, *Caenorhabditis elegans*, drug repositioning, lifespan, longevity, pharmacogenomics

## Abstract

Caloric restriction (CR), a reduction in calorie intake without malnutrition, retards aging in several animal models from worms to mammals. Developing CR mimetics, compounds that reproduce the longevity benefits of CR without its side effects, is of widespread interest. Here, we employed the Connectivity Map to identify drugs with overlapping gene expression profiles with CR. Eleven statistically significant compounds were predicted as CR mimetics using this bioinformatics approach. We then tested rapamycin, allantoin, trichostatin A, LY‐294002 and geldanamycin in *Caenorhabditis elegans*. An increase in lifespan and healthspan was observed for all drugs except geldanamycin when fed to wild‐type worms, but no lifespan effects were observed in *eat‐2* mutant worms, a genetic model of CR, suggesting that life‐extending effects may be acting via CR‐related mechanisms. We also treated *daf‐16* worms with rapamycin, allantoin or trichostatin A, and a lifespan extension was observed, suggesting that these drugs act via DAF‐16‐independent mechanisms, as would be expected from CR mimetics. Supporting this idea, an analysis of predictive targets of the drugs extending lifespan indicates various genes within CR and longevity networks. We also assessed the transcriptional profile of worms treated with either rapamycin or allantoin and found that both drugs use several specific pathways that do not overlap, indicating different modes of action for each compound. The current work validates the capabilities of this bioinformatic drug repositioning method in the context of longevity and reveals new putative CR mimetics that warrant further studies.

## Introduction

The aging of the population has created an urgent need to develop approaches targeting the aging process. In model organisms, aging can be delayed by genetic manipulations and by nutritional interventions like caloric restriction (CR), which consists of restricting calorie intake without malnutrition (Fontana *et al*., 2010; de Magalhães *et al*., 2012). CR is not a perfect solution and comes with several side effects (Dirks & Leeuwenburgh, 2006). Therefore, CR is unlikely to be widely adopted by the public. As such, developing CR mimetics, compounds that reproduce the longevity benefits of CR without its side effects, is of widespread interest.

A number of pathways have been already associated with CR and hence are likely targets of CR mimetics. Target of rapamycin (TOR) is a well‐documented nutrient sensor that regulates metabolism and growth in response to amino acid and nutrient intake (Wullschleger *et al*., 2006; Fontana *et al*., 2010). TOR is inhibited by rapamycin, resulting in an increased lifespan in organisms from yeast to mammals (Stanfel *et al*., 2009; Miller *et al*., 2011). RNAi of TOR increased lifespan in wild‐type *C. elegans*, but not in *eat‐2* mutants, a long‐lived CR genetic model (Hansen *et al*., 2007).

Drug repositioning, finding new applications for known drugs, expedites the drug development process by obviating the need for early toxicity trials. The Connectivity Map (CMap) is a database of gene expression profiles from varying human cell lines responding to treatment with different compounds, including FDA‐approved drugs and nondrug bioactive compounds, which can be used to predict potential small‐molecule candidates (Lamb *et al*., 2006). In this project, we hypothesized that drugs that induce a transcriptional profile in human cell lines overlapping with that of CR are likely to act as CR mimetics. Therefore, we cross‐linked mammalian molecular signatures of CR with drug signatures available in the Connectivity Map and obtained various candidates. Subsequently, we employed lifespan and healthspan assays to test the ability of CR mimetic candidates to induce a CR‐like state in *C. elegans* and found new potential CR mimetics, demonstrating that our method can uncover biologically relevant findings.

## Results

### Cross‐linking CR and drug‐induced transcriptional profiles

To identify potential CR mimetic drugs, we retrieved a CR transcriptional profile from published data in rat cells exposed to sera from rats or rhesus monkeys undergoing CR (de Cabo *et al*., 2003). The transcriptional profile was used to query the CMap to identify drugs that induce similar or opposite profiles (see Experimental procedures). This analysis provided both a list of drugs that match the profile of CR based on all data in the CMap (Table 1) and a list based on different drug dosages for each drug (Table 2). Overall, we discovered 11 small molecules significantly inducing gene expression changes overlapping with those induced by CR. Of note, rapamycin was one of our top hits and is known to extend lifespan in organisms from yeast to mice (Stanfel *et al*., 2009; Miller *et al*., 2011). We also found LY‐294002 to be significant and wortmannin close to significance (Table S2). These molecules also extend lifespan in invertebrates through the inhibition of the PI3K pathway (Babar *et al*., 1999).

**Table 1:** Drugs identified as matching or opposing the transcriptional profile of CR based on all array data

CMap name	Mean	*P*‐value
Geldanamycin	0.325	0
Monorden	0.37	0
Sirolimus	−0.387	0
Allantoin	−0.548	0.00004
LY‐294002	−0.193	0.00004
Rilmenidine	0.619	0.00006
Vorinostat	0.36	0.00014
6‐Bromoindirubin‐3’‐oxime	0.53	0.00022
Antimycin A	−0.517	0.00034

Mean shows the level of match and the direction of the match based on the average of all arrays at all dosages within the CMap; positive values indicate a match, whereas negative ones indicate an opposite profile. Probability values indicate the statistical significance of the match. Only statistically significant drugs after Benjamini correction are shown. Full results are in Table S2.

**Table 2:** Drugs identified as matching or opposing the transcriptional profile of CR based on data from each dose and cell line

CMap name and cell line	Mean	*P*‐value
Sirolimus – MCF7	−0.491	0
Tanespimycin – MCF7	0.378	0
Trichostatin A – PC3	−0.276	0
Geldanamycin – MCF7	0.445	0.00002

Mean shows the level of match and the direction of the match based on the average of all arrays at each dosage, with each cell line, for each drug within the CMap; positive values indicate a match, whereas negative ones indicate an opposite profile. Probability values indicate the statistical significance of the match. Only statistically significant drugs after Benjamini correction are shown. Full results are in Table S2.

### Drug treatments increase lifespan of *C. elegans* in a CR‐like manner

We selected 5 drugs identified as inducing similar or opposite transcriptional profiles to CR to analyse further. These compounds were selected based on different criteria: rapamycin, LY‐294002 and trichostatin A (TSA) because they are already known to increase lifespan; TSA was the top candidate from specific dosages after rapamycin (Table 2); allantoin was chosen as it was a compound not studied previously in the context of aging; and geldanamycin was selected as it induces a transcriptional profile opposite to that of rapamycin and hence would appear to undo CR. To test the selected drugs’ capability to extend lifespan, we performed lifespan assays of wild‐type worms exposed to each drug from the final stage of larval development (after L4 moult, 2 days posthatching). To evaluate whether the mechanism of action of these drugs is similar to that of CR, they were also tested in long‐lived *eat‐2* mutants, as they have a lower food uptake and hence a constant CR‐like state. If these drugs acted through a mechanism different from CR, then we would expect a synergistic increase in lifespan in the *eat‐2* worms. Contrarily, for similar mechanisms, we would expect diminished or no lifespan effects in treated *eat‐2* worms.

**Figure 1:**
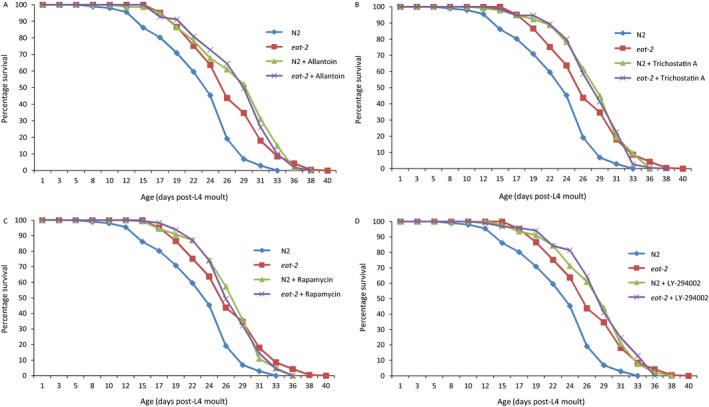
Percentage survival of wild‐type (N2) or *eat‐2* mutant worms alone or treated with allantoin (A), trichostatin A (B), rapamycin (C) or LY‐294002 (D). Worms that did not respond to touch stimulation were recorded as dead. Both *eat‐2* and treated worms of both genotypes showed both a significantly longer lifespan than N2 controls and no significant variation from each other (Table 3). (A) Allantoin‐treated N2 had a 21.9% increase in lifespan. (B) Trichostatin A‐treated N2 had a 22.1% increase in lifespan. (C) Rapamycin‐treated N2 had an 18.9% increase in lifespan. (D) LY‐294002‐treated N2 had a 21.6% increase in lifespan. Results are shown for combined data from two representative trials (full range of results is shown in Table S3).

Allantoin‐ (Fig. 1A), TSA‐ (Fig. 1B), rapamycin‐ (Fig. 1C) and LY‐294002‐treated (Fig. 1D) N2 worms showed a prolonged lifespan compared to N2 controls. The average lifespan of N2 worms treated with allantoin (28.3 days), TSA (28.4 days), rapamycin (27.6 days) and LY‐294002 (28.1 days) was significantly greater than that of untreated controls (23.1 days) (Table 3). This shows that the drugs are sufficient to increase lifespan of wild‐type worms, although the lifespan extension observed in each treatment showed some variation between trials (Table S3). No consistent increase in lifespan was detected in the treated *eat‐2* worms when compared to *eat‐2* controls or to N2 worms treated with the drugs (Fig. 1), although small effects were occasionally observed (Table S3). Taken together, these results indicate that allantoin, TSA, rapamycin and LY‐294002 extend lifespan through a mechanism potentially similar to CR. The exception was geldanamycin‐treated worms (Fig. S1) that did not show any lifespan increase and did not differ significantly from untreated controls of the same genotype. An initial trial with geldanamycin in *eat‐2* worms showed a reduction in lifespan, as if geldanamycin was negating the effect of CR (Table S3). This was not reproduced in further trials.

**Table 3:** Overview of lifespan data for various drug treatments

Name	No. of subjects	Mean lifespan (days)	%Lifespan increase
N2	203	23.14	‐
*eat‐2*	210	27.01	16.22*
N2 + Allantoin	221	28.32	21.86* ^,^ #
*eat‐2* + Allantoin	203	28.45	22.42*
N2 + TSA	277	28.38	22.12*
*eat‐2* + TSA	225	28.22	21.43*
N2 + Geldanamycin	365	23.7	1.98#
*eat‐2* + Geldanamycin	167	28.87	24.23*
N2 + Rapamycin	164	27.64	18.93*
*eat‐2* + Rapamycin	180	28.26	21.60*
N2 + LY‐294002	272	28.09	20.87*
*eat‐2* + LY‐294002	229	28.43	22.33*

The mean number of days each worm population lives post‐L4 moult. These means were compared by a log‐rank test and the results transformed through Bonferroni transformation to compensate for multiple comparisons. *Indicates significance compared to untreated N2 controls; ^#^indicates significance compared to untreated *eat‐2* controls. Results are shown for combined data from two representative trials (full range of results is shown on Table S3). Abbreviations: TSA (trichostatin A).

As *eat‐2* worms eat less, it is possible that they were simply not taking in enough of the drugs to increase lifespan. To this end, we then tested worms with a double dosage for the most novel drugs: allantoin or TSA (Figs S2 and S3). As before, a significant increase in lifespan was observed for all drugs in N2 controls (>20% increase in lifespan, except for the higher allantoin dosage which was only 14%). A statistically significant increase in lifespan was observed when drugs were administered to *eat‐2* worms; however, the effect sizes were very small (<5% increase) and this was observed for both dosages in this trial, suggesting that the much greater lifespan increase in N2 worms is not due to *eat‐2* worms taking up less drug.

Overall, all four drugs predicted to mimic CR (allantoin, TSA, rapamycin and LY‐294002) showed an increase in lifespan in a CR‐like manner. Geldanamycin that showed an opposite gene expression to the above drugs and hence was expected to negate the effects of CR appeared to have no effect on longevity.

### Allantoin and TSA extend lifespan in a DAF‐16‐independent manner

**Figure 2:**
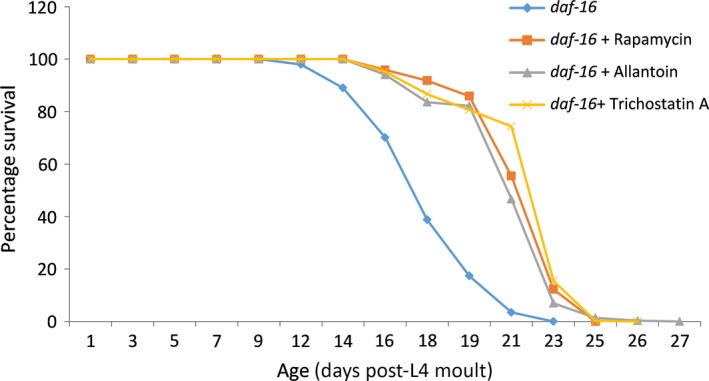
Percentage survival of *daf‐16* worms alone or treated with rapamycin, allantoin or trichostatin A. A significant increase in lifespan was observed in response to treatment with rapamycin (21.7% increase), allantoin (19.7% increase) or trichostatin A (23.8% increase) compared to untreated *daf‐16* worms.

DAF‐16 is not required for lifespan extension induced by CR (Houthoofda *et al*., 2003), but is required for lifespan changes induced by perturbations of the DAF‐2 signalling pathway. If our candidate compounds are CR mimetics, we would expect to see a lifespan increase in *daf‐16* mutant worms. Therefore, we analysed the lifespan of *daf‐16* worms treated with rapamycin, allantoin or TSA. The average lifespan of *daf‐16* worms treated with rapamycin (21.8 days), allantoin (21.5 days) or TSA (22.2) was significantly greater than that of untreated *daf‐16* worms (17.9 days) (Fig. 2), in line with our predictions that these are possible CR mimetics.

### Drug treatments may prolong healthy lifespan

To determine whether the lifespan increase was also affecting healthspan, we measured the healthspan of treated wild‐type worms by performing both movement and pharyngeal pumping assays. Both worm movement and pharyngeal pumping rate have been shown to decrease with age (Hosono *et al*., 1980), and so any delay in this decline would indicate a slower rate of aging. All the treated worms, except those treated with geldanamycin, showed no change in the decline of movement rate (Fig S4–S12).

**Figure 3:**
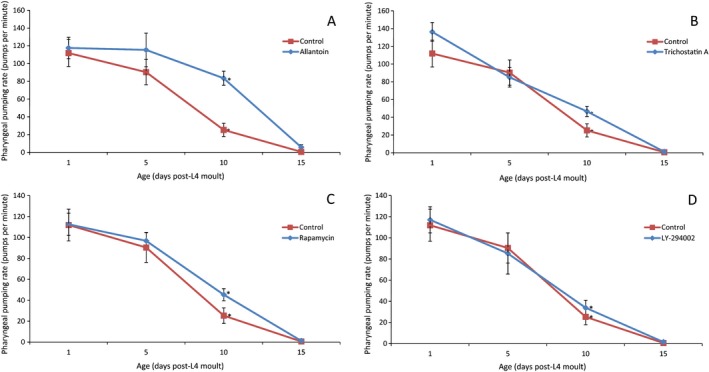
The pharyngeal pumping rate of either N2 control worms or those treated with allantoin (A), trichostatin A (B), rapamycin (C) or LY‐294002 (D). The pumping rate was recorded on days 1, 5, 10 and 15 post‐L4 moult when treatment with the drug began. The treated worms showed a slower decline of pharyngeal pumping compared to the untreated controls. The rates of pumping in allantoin‐ (83.5 pumps per minute), trichostatin A‐ (46.7 pumps per minute), rapamycin‐ (45.3 pumps per minute) and LY‐294002 (33.88 pumps per minute)‐treated worms are greater than those of untreated worms (25.3 pumps per minute) to a statistically significant degree at day 10 (two‐tailed *t*‐test): allantoin (*P* < 0.001, *N* = 10), trichostatin A (*P* < 0.05, *N* = 10), rapamycin (*P* < 0.05, *N* = 10) and LY‐294002 (*P* < 0.01, *N* = 10). *Significance of at least 0.05. Error bars indicate ±1 standard error.

Worms treated with allantoin (Fig. 3A), TSA (Fig. 3B), rapamycin (Fig. 3C) or LY‐294002 (Fig. 3D) showed a significantly slower decline in pharyngeal pumping (Figs S13–S16) and a higher rate of pumping at day 10 when compared to control worms. This difference indicates that the drugs prolong lifespan through delayed aging with worms remaining healthier later in life. It also indicates that drugs are not inducing CR by restricting food intake as the worms’ pharyngeal pumping is not inhibited. At day 15, there is no significant difference between treated and control worms, indicating a faster decline in pumping after day 10 among the treated worms.

**Figure 4:**
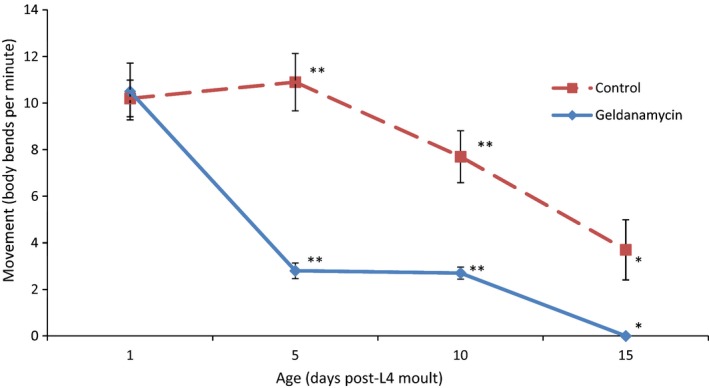
The movement rate of either N2 control worms or those treated with geldanamycin. The movement rate was recorded on days 1, 5, 10 and 15 post‐L4 moult when treatment with the drug began. The geldanamycin‐treated worms show a much faster initial decline in movement compared to the untreated controls before levelling out after day 5 with no decrease between days 5 and 10. Movement rate decreases faster in geldanamycin‐treated worms with the rate of movement at days 5, 10 and 15 for treated worms (2.8, 2.7 and 0 body bends per minute, respectively) being lower than the rate in untreated controls (10.9, 7.7 and 3.7 body bends per minute, respectively), to a significant degree (two‐tailed *t*‐test) at day 5: (*P* < 0.001); day 10: (*P* < 0.001); day 15: (*P* < 0.05). *Significance of at least 0.05; **significance of at least 0.005. Error bars indicate ±1 standard error.

Geldanamycin‐treated worms showed a faster decline of pharyngeal pumping rate compared to untreated controls. The rate of pumping in treated worms was lower at day 5 (Fig. S13). Movement rate decreased faster in geldanamycin‐treated worms (Fig. 4). The rate of this decline is much greater between days 1 and 5; however, the rate seems to level off. These results indicate that the worms are aging at a faster rate or are suffering some toxic effect of the drug, despite the lack of reduction in lifespan.

### Analysis of predicted drug targets and impact on CR and longevity networks

To explore the mechanisms and pathways by which the tested drugs mimic CR, we looked at their predicted targets. Using STITCH (Kuhn *et al*., 2012), we identified 5 gene targets for allantoin, 19 for rapamycin, 5 for LY‐294002 and 9 targets for TSA. Although the overlap between the four target sets was minimal, with only 2 genes being common targets for rapamycin and for LY‐294002 (*let‐363*, a homologue of *mTOR*, and *rps‐6*), it is still possible that all mechanisms converge to a downstream signature. This idea is further supported by our MANTRA (Carrella *et al*., 2014) analysis, which allows the visualization of the similarity in induced gene expression for a set of drugs using a network (Fig. S17). This analysis showed that geldanamycin, rapamycin and LY‐294002 act in a relatively similar manner, with TSA having a more distant profile. Allantoin seems to act in a manner distinct from the rest of the compounds as it appears unconnected to the other compounds (Fig. S17).

**Figure 5:**
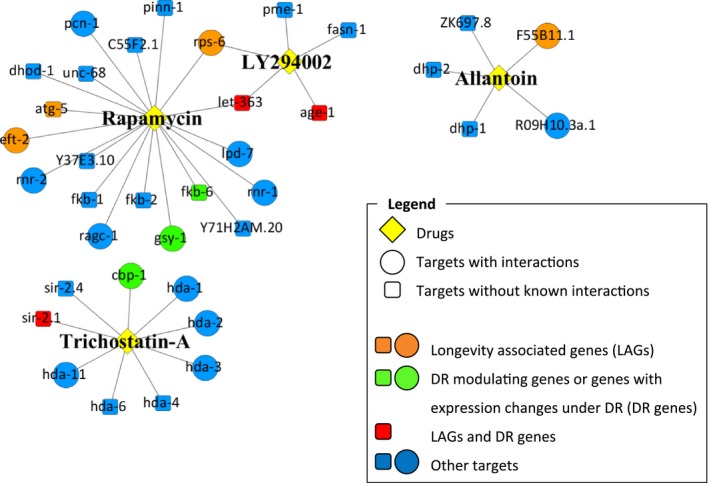
Visual representation of allantoin, LY‐294002, rapamycin and trichostatin A and their gene/protein targets. Included is the mapping of longevity‐associated genes, CR‐mediating genes and genes with expression changes under CR.

Next, we aimed to understand the links between the targets of the drugs, longevity and CR (Fig. 5). Using the GenAge database (Tacutu *et al*., 2013), we found among the drug targets genes previously associated with lifespan regulation in worms. For example, RNAi of *let‐363* and *rps‐6* (rapamycin and LY‐294002 targets), and RNAi of *F55B11.1* (one of the predicted targets of allantoin), extends lifespan of N2 worms. In contrast, RNAi of *atg‐5* and postdevelopmental RNAi of *eef‐2*, both potential targets of rapamycin, result in a lifespan reduction. Other targets include *age‐1* (target of LY‐294002), whose mutants are well known to be long lived, and *sir‐2.1* (target of TSA) whose overexpression extends lifespan by up to 50%. Interestingly, *let‐363*,* rps‐6* and *sir‐2.1* are also in GenDR as associated with CR longevity effects (Wuttke *et al*., 2012). Additionally, we found three genes so far not associated with longevity which have been suggested to play a role in CR: orthologs of *fkb‐6* and *gsy‐1* (both targets of rapamycin) are significantly overexpressed in mice under CR (Plank *et al*., 2012). Additionally, cbp‐1 (a transcriptional factor that might be affected by TSA) is induced under CR and blocking it results in countering the protective effects of CR (Zhang *et al*., 2009).

**Figure 6:**
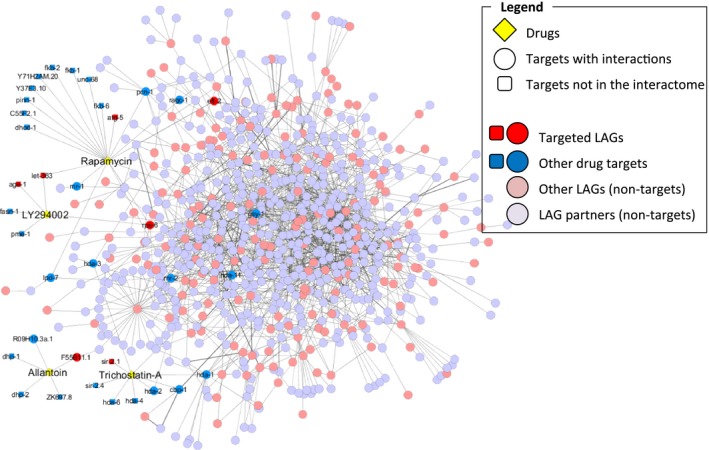
The worm longevity network. The network (which includes longevity‐associated genes and their interaction protein partners) contains many potential links to drug targets through which allantoin, LY‐294002, rapamycin and trichostatin A might have an effect on its functionality. For an integrative visualization, all drug targets have been displayed (including the targets that do not belong to the network).

As previously shown, all longevity‐associated genes (LAGs), together with their direct interaction partners, form a continuous protein–protein interaction network. This worm longevity network was successfully used to predict new longevity regulators, and we have shown that the first‐order interactors of known worm LAGs are also likely to participate in the regulation of lifespan (Tacutu *et al*., 2012). As such, it is interesting to know how many more non‐LAG targets belong to the worm longevity network (Fig. 6). Of the 11 additional targets, 8 have multiple interactions, some being quite central. For example, *hda‐1*, one of TSA targets, interacts with 15 other network genes, and *gsy‐1* and *pcn‐1*, both targets of rapamycin, interact with 11 and 9 other genes, respectively. This suggests that TSA and rapamycin might modulate lifespan in a systemic way. While allantoin and LY‐294002 are more peripheral in the network, some of their targets are also not very far from central nodes and LAGs. Thus, although the targets for the drugs hardly overlap, it seems that these four drugs perturb the longevity network, either by targeting specific genes or through effects on multiple genes that are different between the drugs, to modulate longevity.

### Gene expression analysis of rapamycin and allantoin

Rapamycin, thought to be a CR mimetic, is one of the most studied drugs, and understanding this compound better is essential to better understanding CR. To this end, we decided to evaluate gene expression changes in both *eat‐2‐* and N2‐treated worms. Allantoin, on the other hand, has not been studied in terms of aging or CR. As such, its analysis in N2 worms is also important to understand whether it acts similarly to rapamycin and/or to CR in *eat‐2* mutants.

The number of differentially expressed genes (DEGs) (*P*‐value <0.05 and fold change >2.0) across treatments is presented in Table 4 (full list in Table S4). Rapamycin‐treated N2 worms showed a large number of changes (vs. N2 controls) when compared to allantoin‐treated N2 worms, *eat‐2* control and rapamycin‐treated *eat‐2* worms (Table 4).

**Table 4:** Summary of differentially expressed genes (*P*‐value <0.05, fold change >2)

	N2 Allantoin		N2 Rapamycin		*eat‐2* Control		*eat‐2* Rapamycin	
N2 Control	77↑	287↓	907↑	672↓	116↑	93↓	216↑	409↓
N2 Allantoin			2706↑	1978↓	346↑	243↓	197↑	55↓
N2 Rapamycin					1487↑	2178↓	1701↑	2613↓
*eat‐2* Control							238↑	312↓

Comparisons are based on the set defined at the top of the table vs. the set defined on the leftmost column.

First, we wanted to determine whether the drugs affect a large portion of genes in N2 worms. Feeding allantoin or rapamycin to worms results in several hundred genes being differently expressed, both up‐ and downregulated, with rapamycin having a wider effect. This suggests that the effect of both drugs is not localized to a small set of targeted genes, but results in widespread changes. Interestingly, the signatures produced by rapamycin and allantoin are drastically different. While allantoin downregulates many genes (almost 80%), for rapamycin more than 55% of the affected genes are upregulated. Furthermore, the overlap of genes changing in the same direction is low (upregulated genes: not significant, Fisher's one‐sided, *P* = 0.37; downregulated genes: lower than expected, *P* = 1.6E‐3). The difference can also be observed in terms of functional enrichment (Table S5). For allantoin, we found only that ‘neuropeptide signalling pathway’ and ‘fatty acid metabolism’ are enriched in downregulated genes, while rapamycin affects several biological processes: moulting cycle, dephosphorylation, regulation of multicellular organism growth, cuticle development and body morphogenesis are enriched in upregulated genes, and embryonic development, cell cycle and nuclear division, cell fate, cellular response to various stresses, negative regulation of metabolic processes and biosynthesis, TGFb and WNT signalling are all enriched in downregulated genes. Many of these findings (e.g. downregulation of cell cycle‐related terms) are in accordance with current knowledge regarding the effects of rapamycin. While our analysis focused mostly on biological processes and pathways, both up‐ and downregulated genes included previously established LAGs, which is in line with the above results from potential drug targets that suggest effects at the level of the longevity network (Table S6).

Next, we wished to determine whether the lack of lifespan effect of rapamycin in *eat‐2* mutants can be seen at a transcriptional level. The functional comparison of *eat‐2* and N2 controls revealed only a few enriched terms (body morphogenesis, sphingolipid metabolism, lipase activity and aspartic‐type peptidase activity; Table S5), in line with the low number of DEGs (Table 4). Still, the profile of rapamycin‐treated *eat‐2* worms shows a smaller number of DEGs (238 up, 312 down), a result very different than for rapamycin‐treated N2 worms (>1500 DEGs). While there is a fair amount of overlap between the two rapamycin profiles: 44 genes in the same direction changes (up: *P* = 3.39E‐5; down: *P* = 1.10E‐3), there are also 35 genes with opposite changes (more than expected by chance). In contrast to the functional analysis in the rapamycin‐treated N2 worms, in the *eat‐2* background a much smaller number of terms are enriched. Interestingly, one of the functional categories enriched with downregulated genes is regulation of growth and determination of adult lifespan, although we have not observed any noticeable phenotype in the treated *eat‐2* worms.

Lastly, we aimed to assess whether the pattern produced by allantoin or rapamycin in N2 worms is similar to that of *eat‐2* controls. Allantoin‐treated N2 worms have ~600 DEGs (346 up, 243 down) compared to *eat‐2* controls, while rapamycin changes more than 3500 genes (1487 up, 2178 down). Comparing the relative changes caused by allantoin in N2 worms with the changes between *eat‐2* and N2, there is a significant overlap among downregulated genes (16 genes, Fisher's one‐sided, *P* = 4.69E‐14), but not for upregulated genes. Contrarily, rapamycin‐induced expression changes that overlap with *eat‐2* vs. N2 changes have predominantly opposite directions (56 genes; *P* < 2.59E‐08).

## Discussion

The fact that rapamycin was one of the top results among over 1000 drugs tested using our drug repositioning approach is a strong indicator that this bioinformatics approach provides biologically relevant results. This is further strengthened by the presence of other known life‐extending compounds such as LY‐294002 and wortmannin (Babar *et al*., 1999). We have shown that rapamycin, LY‐294002, TSA and allantoin can increase lifespan in *C. elegans* in a manner that does not act synergistically with CR‐induced lifespan extension, indicating that these compounds and CR may act through a similar mechanism. Additionally, we have shown that this lifespan extension comes with a possible slowing of aging seen through a moderately slower decline in pharyngeal pumping rate, but with no change in movement rate decline.

Previous studies have shown that rapamycin increases lifespan of N2 worms by 19% (Robida‐Stubbs *et al*., 2012), which is similar to our results despite their use of 10 times the dose used in this study (100 μm). These results help indicate the reliability of the data collected. Rapamycin acts via the TOR pathway (Stanfel *et al*., 2009), which is thought as crucial in CR. DAF‐16 is not required for the lifespan increase induced by rapamycin (Fig. 4), as expected.

LY‐294002 is a known inhibitor of phosphatidylinositol 3‐kinase (age‐1) and has been shown to increase lifespan in adult worms by ~10% (Babar *et al*., 1999). This is slightly lower than observed in the present study. The difference between our study and the Babar *et al*.'s research could be the result of a higher dose of LY‐294002 at 1.6 mm by Babar *et al*., which may be having a toxic effect. This is supported by Barbar *et al*.'s use of a higher dose at 2.4 mm, which showed no significant increase in lifespan. Mutations of *age‐1* and *daf‐2*, which acts upstream of *age‐1*, result in lifespan extension in N2 worms, but *daf‐2* mutations and RNAi also increases the lifespan of *eat‐2* worms, which suggests that *age‐1* would do so also (Chen *et al*., 2007). This is the opposite of what we have found. This may be due to the drug treatment not completely inhibiting *age‐1*. AGE‐1 acts upstream of TOR, and hence, the connection between LY‐294002 treatment and CR becomes clear.

Allantoin has not been previously studied in terms of effects on aging. It has been shown to bind to imidazoline receptors in mammals and have beneficial effects on energy regulation, potentially through the activation of AMP kinase which could then act through the mTOR pathway (Chen *et al*., 2012). As *C. elegans* does not appear to contain any imidazoline receptors, it is unclear how allantoin produces the effects seen in this study. It is still possible that a similar pathway exists in worms acting through yet undiscovered receptors. As allantoin is likely produced in *C. elegans* as a by‐product of the reaction of uric acid and reactive oxygen species (Artyukhin *et al*., 2013), there is the potential for a feedback mechanism by which allantoin signals the presence of oxidative stress. This stress signal would then activate stress response pathways similar to those activated under CR to give the observed results. Our results show that DAF‐16 is not required for the lifespan increase induced by allantoin.

TSA is a well‐documented inhibitor of class 1 and 2 HDACs and hence has the potential to induce widespread epigenetic changes. TSA has been shown to extend lifespan in *C. elegans* by about 11% (Zhang *et al*., 2009), which is in line with our results (Table S3). One possible mechanism to explain TSA's lifespan extension effects is through the production of nitric oxide (NO). TSA has been shown chemically to produce NO when it is oxidized (Samuni *et al*., 2010) and hence could potentially produce NO in worms. *C. elegans* have been shown to live longer when fed NO‐producing bacteria in a process that results in hsp16 activation (Gusarov *et al*., 2013). Increases in the heat shock protein hsp16 have been shown to increase lifespan in *C. elegans* in a DAF‐16‐dependent manner (Walker & Lithgow, 2003). However, DAF‐16 is not required for the lifespan increase induced by TSA. As such, further mechanistic studies are warranted to identify how TSA increases lifespan.

At the given dose, geldanamycin was unable to affect lifespan of wild‐type or *eat‐2* worms. However, N2 worms showed a significant increase in the rate of decline of both movement and pharyngeal pumping compared to untreated controls, suggesting that the drug is having a biological effect. Geldanamycin, unlike the other drugs in this study, was predicted to induce a transcriptional profile opposite to that of rapamycin. Another study showed that prolonged treatment of geldanamycin had detrimental effects on motility and is hence in line with our results (Skantar *et al*., 2005). Geldanamycin inhibits Hsp90, mutants of which have been shown to have muscle cell defects (Gaiser *et al*., 2011). One of our trials indicated that geldanamycin prevented the lifespan extension in *eat‐2* worms; however, these results were not reproducible.

We have observed a correlation between increased lifespan and prolonged pharyngeal pumping in treated worms. However, movement rate was unaffected even in long‐lived worms with prolonged pharyngeal pumping. This indicates that either movement rate is a less sensitive measure of aging rate than pharyngeal pumping or that movement rate and pharyngeal pumping rate decline are delayed by two different mechanisms. Those drugs that delayed the decline of pharyngeal pumping did so in a manner that was only seen significantly at day 10. It is likely that such observation could not be seen at earlier time points because the pumping rate does not decline much until after day 5, so no difference could be seen between the control worms and the apparently slow‐aging treated worms. The reason why there is no observed difference after day 10 is unclear. Perhaps the controls are declining to the low level seen at day 15 earlier and the pumping rate is remaining constant, or that the long‐lived treated worms have a faster decline that starts later in life.

To address the possibility that *eat‐2* worms are not taking up as much of the drugs as N2, we retested our most interesting drugs using a double dosage. *Eat* mutant worms have been shown to have a pharyngeal pumping rate of 20% that of wild‐type worms, but a more recent study measuring food uptake suggests that they consume 60–80% as much food as wild‐type worms (Gomez‐Amaro *et al*., 2015). As such, using double the drug concentrations should allow *eat‐2* mutants to uptake at least as much drug as N2. Because we obtained equivalent results with a higher dosage, this suggests that our drugs may indeed be acting *via* CR mechanisms. Further dosages in worms and in other model organisms of all the identified drugs should be studied in future.

To obtain a better understanding of the changes that the studied drugs cause in worms, we analysed the differences in gene expression in response to allantoin and rapamycin. While both drugs affect large numbers of genes in the N2 strain, rapamycin has a much wider effect and the signatures produced by rapamycin and allantoin were quite different. Nevertheless, both drugs extend lifespan and, as can be seen from the worm longevity network (Fig. 6), interact with longevity‐associated genes and their partners. As such, it is possible that a common downstream mechanism for the two drugs exists, only triggered through different signals and pathways. Our analysis of potential targets of the four life‐extending drugs (Fig. 5) supports this view.

The number of rapamycin‐affected genes is much smaller in the *eat‐2* background (Table 4), compared to the wild‐type N2, which probably reflects the lack of an observable phenotype. It is unclear, however, whether this is due to rapamycin having a background‐specific effect, or because *eat‐2* mutants eat less, and thus, the dose of rapamycin is reduced as well. Neither allantoin nor rapamycin produced a gene expression pattern highly similar to that of *eat‐2* controls. A potential caveat is that the effects at other drug dosages have not been investigated. Different doses can have different effects, as seen in a recent study using the supposed CR mimetic metformin, in which some doses increased N2 lifespan, but decreased *eat‐2* lifespan (Onken & Driscoll, 2010). However, regardless of this caveat, we have shown that the drugs computationally predicted to act as CR mimetics seem to do so at the tested dosages.

Pathways identified to affect aging may or may not be conserved between the nematode worm *C. elegans* and mammals and physiological differences could also affect how the drugs act (de Magalhães, 2014). It is important then to test whether the effects of these drugs are conserved in mammals such as mice. While further experiments for the effect of these drugs in mammals are undoubtedly necessary, our experiment also provides some promising hints towards these mechanisms being evolutionary conserved. The initial gene expression profile used as input for CMap was obtained from rodent cells exposed to sera from animals (rats or rhesus monkeys) under CR and was then compared with drug‐induced expression profiles in human cells. Testing these drugs in worms showed an extended longevity phenotype, suggesting that their CR mimetic effects may be evolutionary conserved.

## Conclusions

Bioinformatic approaches to discover drugs capable of delaying aging have been suggested (Fortney *et al*., 2012; de Magalhães *et al*., 2012) and have proven fruitful in the past, albeit using different methodologies (Ye *et al*., 2014). We have identified four compounds that result in both lifespan and healthspan extension in a manner we would expect to see from CR. These drugs include known CR mimetics but also allantoin, a compound that, to our knowledge, has not yet been studied in the context of longevity. Our analysis of CR and longevity networks also suggests that while these compounds initially target different genes in the networks, they may converge on common downstream CR‐related pathways modulating longevity. Overall, the present study serves as proof of concept that bioinformatics drug repositioning approaches can identify and prioritize putative CR mimetics, and reveals new candidate compounds that warrant further studies.

## Experimental procedures

### Connectivity Map analysis

A list of 41 DEGs from rat cell lines exposed to Fisher 344 rat and rhesus monkey sera from CR states (compared to cells exposed to sera from *ad libitum* animals) was collected from the literature (de Cabo *et al*., 2003). Using HomoloGene (NCBI Resource Coordinators 2014), only genes with human homologues were kept (39 genes in total).

The obtained geneset (Table S1) was used in NetAffx probe batch query (Liu *et al*., 2003), and the identified probes were then used to query the Connectivity Map (Lamb *et al*., 2006) to obtain data on the 1309 small molecules in the database. To account for multiple hypothesis testing, Benjamini correction (Benjamini & Yekutieli, 2001) was performed with a 0.05 *P*‐value cut‐off (Table S2). The output includes potential CR mimetics, such as rapamycin, and drugs known to extend lifespan, like LY‐294002, with negative scores, and hence, we treated negative scoring as a match for the profile. We have no explanation why negative scores should be a stronger indication of potential CR mimetics, but as mentioned above we performed our follow‐up on three other drugs (LY‐294002, TSA and allantoin) with profiles in the same direction as rapamycin and one drug (geldanamycin) with an opposite profile.

### Worm strains and cultures

N2 wild isolate strain, *eat‐2* (DA465 strain) and the *daf‐16* (mgDf50) mutants were used in this study. All worm strains were provided by the *Caenorhabditis Genetics Centre* (CGC). The *eat‐2* mutants have reduced neuronal signalling due to the loss of the Eat‐2 nicotinic acetylcholine receptor resulting in decreased pharyngeal pumping that induces a CR‐like state by restricting feeding. All strains were grown at 20 °C on standard nematode growth medium (NGM) as described in ‘WormBook’ (Stiernagle, 2006) and fed *Escherichia coli (E. coli)* OP50 unless otherwise stated.

Killed *E. coli* OP50 was used as the worm food source for the lifespan and healthspan assays from L4 moult onwards. Saturated OP50 stock was pelleted and ethanol was added to make a 20% ethanol solution. This solution was irradiated with UV radiation in a Bio‐Rad GS gene linker set at 0–999 mJ/cm^2^ power 10 times to finalize the killing of the *E. coli*, which was pelleted and resuspended in LB broth.

### Drug concentrations

Rapamycin (ref. R0395), geldanamycin (ref. G3381), allantoin (ref. 93791) and FUDR (ref. F0503) were purchased from Sigma Aldrich, Dorset, and LY‐294002 (ref. 9901S) and TSA (ref. 9950S) were purchased from New England Biolabs, Hitchin. The drug under study was added to 3 mL NGM to give the following final concentration: 10 μm rapamycin, 20 μm geldanamycin, 100 μm LY‐294002, 100 μm TSA and 250 μm allantoin. These concentrations were determined by taking values seen in previous studies in worms and invertebrates and also by treating worms with variations of this dose and monitoring for increased mortality. The highest concentration used in a preliminary experiment (not shown) that did not result in increased worm mortality was used for the study; however, it was later found that for some drugs the induced mortality was the result of increased levels of dimethyl sulphoxide (DMSO), not the drugs themselves, and hence the dosages used may be lower than the maximum nonlethal dose. A double dose of TSA (200 μm) and allantoin (or 500 μm) was also analysed.

### Lifespan assays

N2, *daf‐16* and *eat‐2* worms were synchronized as described previously (Stiernagle, 2006) and grown as described above until they reached the L4 moult. L4 worms were used to allow for the use of FUDR and easier comparison to established literature. N2, *daf‐16* or *eat‐2* worms were then transferred to six‐well plates containing 3 mL NGM that contained 30 μL FUDR (10 gr L^−1^) in dH_2_O, and the drug of study dissolved in 100 μL DMSO or 100 μL DMSO (final concentration 0.14M) alone in control treatments. Worms were counted at least three times a week, and dead animals (unresponsive to touch stimuli) were removed; 40 μL of killed OP50 was added three times a week to all plates until day 15 of the assay by which point the rate of food consumption was so low that no additional food was required. Wells had 100 μL dH_2_O added as needed after day 15 to prevent the plates from drying out. See Table S7 for tabulated lifespan data.

### Healthspan assays

Movement and pharyngeal pumping assays were performed as described by Huang *et al*. (2004), except that the N2 worms used were selected at random from the populations within the lifespan assay. For the movement assay, the plates were firmly tapped onto the microscope to stimulate movement. Body bends (defined as the movement of the head in a half sigmoidal wave that produced forward movement and/or resulted in a bend in the body that moved halfway down the length of the worm) were counted for a minute for 10 worms at 1, 5, 10 and 15 days post‐L4 moult. For pharyngeal pumping assays, undisturbed worms (before movement assay) were monitored for a minute and pumps counted for 10 worms at 1, 5, 10 and 15 days post‐L4 moult.

### Statistical analysis

Lifespan assays (Table S3) and healthspan assays (Figs S4–S16, S18 for trials not shown in this paper) were repeated at least twice. Log‐rank tests and mean lifespan calculations were performed using OASIS (Yang *et al*., 2011). Two‐tailed *t*‐tests of healthspan data were analysed using Matlab after normality testing by Kolmogorov–Smirnov test. Statistical analysis of geneset overlaps was carried out using one‐sided Fisher's exact test, using r (R Core Team, Vienna, Austria).

### Bioinformatics analysis

Potential drug targets for allantoin, LY‐294002, rapamycin and TSA were retrieved from the STITCH database, version 3.1 (Kuhn *et al*., 2012). The comparison of drug targets with known worm LAGs and with genes implicated in CR was made using data from the GenAge database (build 16) and the GenDR database (build 1) – both available through the Human Ageing Genomic Resources (Tacutu *et al*., 2013). Cross‐species gene comparisons (e.g. worm drug targets that have mouse orthologs differentially expressed under CR conditions) were made using orthologs from the InParanoid7 database (Ostlund *et al*., 2010), with exclusion of in‐paralogs for scores <0.05.

Protein–protein interaction data were retrieved from the BioGRID database (Stark *et al*., 2011), release 3.1.83. The construction of longevity networks has been described previously (Tacutu *et al*., 2010). Briefly, the networks include LAGs as a core set and their first‐order interaction partners. Only the largest connected component is kept in the network. The graphical output for the networks was generated using cytoscape 3.0.1 (Smoot *et al*., 2011).

### Gene expression analysis

Gene expression analysis was performed 72 h after the worms were transferred to drug‐containing plates. The transfer was performed when worms were in the L4 stage. Whole worms were washed from the plate with distilled water and spun down at 3000 rpm (∼750 g) for 2 min. Afterwards, the worms were resuspended in 400 uL Trizol reagent and then transferred to 2‐mL screw cap tubes containing 1 mL 0.1‐mm glass beads. Worms were lysed in a powerlyser at 4000 rpm (∼1300 g) for two 30‐second runs, chilling the tubes on ice after each run. RNA was then extracted as per the manufacturer's instructions with a QIAGEN RNeasy extraction kit (Qiagen, Venlo, The Netherlands). Each condition was run in triplicate.

RNA samples were analysed in terms of quality and quantity using an Agilent 2100 Bioanalyser RNA 6000 Nano chip. Samples were labelled using the Affymetric GeneChip 3@ IVT Express labelling kit as per the manufacturer's instructions; 200 ng of total RNA was input into the labelling reaction. Following amplification, 15ug of RNA was fragmented and 12.5ug of fragmented labelled RNA was hybridized onto Affymetrix GeneChip *C. elegans* Genome Arrays as per the manufacturer's instructions. These were hybridized for 16 h at 45 °C and 60 rpm in an Affymetrix Hybridization Oven 640. Hybridized arrays were washed using Affymetrix Hybridization Wash and Stain kit on a GeneChip Fluidics station 450 and scanned using an Affymetrix GeneChip scanner 3000 7G. Affymetrix GeneChip Command Console Software was used to generate CEL files. Raw data have been submitted to Gene Expression Omnibus (GEO) under the accession GSE64336.

Analysis of gene expression data was carried out with the Affymetrix^®^ Transcriptome Analysis Console. Data preprocessing (using RMA normalization) and QC metrics were performed using Affymetrix^®^ Expression Console^™^ and manually inspected afterwards (see Fig. S19 for array heat map). Expression analysis was carried out for each two pairwise conditions. FDR statistical correction for multiple testing resulted in a slightly lower number of DEGs in most cases. The notable exceptions to this pattern are the comparisons involving N2 controls that appear to be affected more by noise. This is most probably due to a higher variability in gene expression in the N2 control population compared to populations with pathological or drug‐induced conditions. For example, surveying various worm data sets from experiments submitted to GEO revealed that, in many cases, there is a striking number of genes differentially expressed between N2 worm samples from different experiments (data not shown). To include DEGs that are only marginally significant in our subsequent analysis, we have included all DEGs with *P*‐value <0.05 and fold change >2.0. This is in line with our previous methods that focussed on finding pathways potentially associated with aging and longevity (de Magalhães *et al*., 2009). DEGs were matched up against known worm LAGs and against genes implicated in CR using data from the GenAge and GenDR databases (Tacutu *et al*., 2013).

Functional enrichment analysis was performed for DEGs with the tools provided by DAVID 6.7 (Huang *et al*., 2009). For each analysis, the worm genome was used as background with default settings. Multiple hypothesis testing was carried out using Benjamini correction (*P* < 0.05). For each data set enriched in more than 5 terms, functional annotation clustering was carried out, using default parameters and an EASE enrichment threshold of 0.05. Terms that did not fit our criterion (Benjamini *P* < 0.05) were removed postanalysis from the constructed clusters.

## Funding info

This project was supported by a Royal Society grant (RG120543) to JPM. SC is supported by a BBSRC DTP studentship (BB/J014516/1). RT was supported by a Marie Curie FP7‐PEOPLE‐IEF Fellowship within the 7th European Community Framework Programme.

## Conflict of interest

None declared.

## Supporting information


**Fig. S1** Percentage survival of wild‐type (N2) or *eat‐2* mutant worms alone or treated with geldanamycin.
**Fig. S2** Percentage survival of wild‐type (N2) or *eat‐2* mutant worms alone or treated with a regular or double dose of trichostatin A (TSA).
**Fig. S3** Percentage survival of wild‐type (N2) or *eat‐2* mutant worms alone or treated with a regular or double dose of allantoin.
**Fig. S4** The movement rate measured as the number of body bends per minute of either N2 control worms or those treated with allantoin, trial 1.
**Fig. S5** The movement rate measured as the number of body bends per minute of either N2 control worms or those treated with allantoin, trial 2.
**Fig. S6** The movement rate measured as the number of body bends per minute of either N2 control worms or those treated with trichostatin A, trial 1.
**Fig. S7** The movement rate measured as the number of body bends per minute of either N2 control worms or those treated with trichostatin A, trial 2.
**Fig. S8** The movement rate measured as the number of body bends per minute of either N2 control worms or those treated with rapamycin, trial 1.
**Fig. S9** The movement rate measured as the number of body bends per minute of either N2 control worms or those treated with rapamycin, trial 2.
**Fig. S10** The movement rate measured as the number of body bends per minute of either N2 control worms or those treated with LY‐294002, trial 1.
**Fig. S11** The movement rate measured as the number of body bends per minute of either N2 control worms or those treated with LY‐294002, trial 2.
**Fig. S12** The movement rate of either N2 control worms or those treated with geldanamycin, trial 2.
**Fig. S13** The Pharyngeal Pumping rate of either N2 control worms or those treated with geldanamycin.
**Fig. S14** The Pharyngeal Pumping rate of either N2 control worms or those treated with allantoin, trial 2.
**Fig. S15** The Pharyngeal Pumping rate of either N2 control worms or those treated with trichostatin A, trial 2.
**Fig. S16** The Pharyngeal Pumping rate of either N2 control worms or those treated with Rapamycin, trial 2.
**Fig. S17** Mantra 2.0 analysis of the compounds under study.
**Fig. S18** The Pharyngeal Pumping rate of either N2 control worms or those treated with LY‐294002, trial 2.
**Fig. S19** Pearson's correlation of the signal (using RMA‐normalized gene‐level signals) between samples, obtained from the Affymetrix Expression Console.

**Table S1** Human genes used to query the Connectivity Map and their relative change in the CR transcriptional profile used.
**Table S3** The variation seen in lifespan compared to controls of each treatment.

**Table S2** Full list of results from the Connectivity Map. Specificity score gives an estimate of how unique the similarities of the drugs’ expression profile to the query expression profile are.

**Table S4** Full list of differentially expressed genes.

**Table S5** Functional enrichment for genes differentially expressed under various treatments.

**Table S6** List of differentially expressed longevity‐associated genes.

**Table S7** Tabulated lifespan data for Figures 1, 2, S1, S2 and S3.
