# Supplementary material for: A network pharmacology approach reveals new candidate caloric restriction mimetics in C. elegans
Source: Aging Cell. 2015 Dec 16;15(2):256–66. doi: 10.1111/acel.12432 (PMC4783339; doi:10.1111/acel.12432)
Supplement: Supplementary file 1 — Fig. S1 Percentage survival of wild‐type (N2) or eat‐2 mutant worms alone or treated with geldanamycin. Fig. S2 Percentage survival of wild‐type (N2) or eat‐2 mutant worms alone or treated with a regular or double dose of trichostatin A (TSA). Fig. S3 Percentage survival of wild‐type (N2) or eat‐2 mutant worms alone or treated with a regular or double dose of allantoin. Fig. S4 The movement rate measured as the number of body bends per minute of either N2 control worms or those treated with allantoin, trial 1. Fig. S5 The movement rate measured as the number of body bends per minute of either N2 control worms or those treated with allantoin, trial 2. Fig. S6 The movement rate measured as the number of body bends per minute of either N2 control worms or those treated with trichostatin A, trial 1. Fig. S7 The movement rate measured as the number of body bends per minute of either N2 control worms or those treated with trichostatin A, trial 2. Fig. S8 The movement rate measured as the number of body bends per minute of either N2 control worms or those treated with rapamycin, trial 1. Fig. S9 The movement rate measured as the number of body bends per minute of either N2 control worms or those treated with rapamycin, trial 2. Fig. S10 The movement rate measured as the number of body bends per minute of either N2 control worms or those treated with LY‐294002, trial 1. Fig. S11 The movement rate measured as the number of body bends per minute of either N2 control worms or those treated with LY‐294002, trial 2. Fig. S12 The movement rate of either N2 control worms or those treated with geldanamycin, trial 2. Fig. S13 The Pharyngeal Pumping rate of either N2 control worms or those treated with geldanamycin. Fig. S14 The Pharyngeal Pumping rate of either N2 control worms or those treated with allantoin, trial 2. Fig. S15 The Pharyngeal Pumping rate of either N2 control worms or those treated with trichostatin A, trial 2. Fig. S16 The Pharyngeal Pumping rate of either N2 contr [file ACEL-15-256-s001.pdf]

Supporting Information

Calvert et al., ***A network pharmacology approach reveals new candidate caloric restriction mimetics***

Table of Contents:

SuppInfo 1.pdf:

Table S1. Human genes used to query the Connectivity Map and their relative change in the CR transcriptional profile used.

Table S3. The variation seen in lifespan compared to controls of each treatment.

Figure S1. Percentage survival of wild-type (N2) or *eat-2* mutant worms alone or treated with geldanamycin.

Figure S2. Percentage survival of wild-type (N2) or *eat-2* mutant worms alone or treated with a regular or double dose of trichostatin A (TSA). N2 worms treated with either 100µM or 200µM of TSA lived significantly longer than N2 (22.6% and 23.8%) and *eat-2* worms (6% and 7%). Treatment of *eat-2* worms with either dose led to a statistically significant increase in lifespan, however this was only a small increase (4.2% and 3.6%).

Figure S3. Percentage survival of wild-type (N2) or *eat-2* mutant worms alone or treated with a regular or double dose of allantoin. N2 worms treated with either 250µM or 500µM of allantoin lived significantly longer than N2 (20.1% and 14.3%). N2 worms treated with 250µM of allantoin but not 500µM was significantly longer lived than *eat-2* worms (3.9%). Treatment of *eat-2* worms with either dose led to a statistically significant increase in lifespan, however this was only a small increase (4.2% and 3.3%).

Figure S4. The movement rate measured as the number of body bends per minute of either N2 control worms or those treated with allantoin, trial 1.

Figure S5. The movement rate measured as the number of body bends per minute of either N2 control worms or those treated with allantoin, trial 2.

Figure S6. The movement rate measured as the number of body bends per minute of either N2 control worms or those treated with trichostatin A, trial 1.

Figure S7. The movement rate measured as the number of body bends per minute of either N2 control worms or those treated with trichostatin A, trial 2.

Figure S8. The movement rate measured as the number of body bends per minute of either N2 control worms or those treated with rapamycin, trial 1.

Figure S9. The movement rate measured as the number of body bends per minute of either N2 control worms or those treated with rapamycin, trial 2.

Figure S10. The movement rate measured as the number of body bends per minute of either N2 control worms or those treated with LY-294002, trial 1.

Figure S11. The movement rate measured as the number of body bends per minute of either N2 control worms or those treated with LY-294002, trial 2.

Figure S12. The movement rate of either N2 control worms or those treated with geldanamycin, trial 2.

Figure S13. The Pharyngeal Pumping rate of either N2 control worms or those treated with geldanamycin.

Figure S14. The Pharyngeal Pumping rate of either N2 control worms or those treated with allantoin, trial 2.

Figure S15. The Pharyngeal Pumping rate of either N2 control worms or those treated with trichostatin A, trial 2.

Figure S16. The Pharyngeal Pumping rate of either N2 control worms or those treated with Rapamycin, trial 2.

Figure S17. Mantra 2.0 analysis of the compounds under study.

Figure S18. The Pharyngeal Pumping rate of either N2 control worms or those treated with LY-294002, trial 2.

Figure S19. Pearson's correlation of the signal (using RMA-normalized gene-level signals) between samples, obtained from the Affymetrix Expression Console.

SuppInfo 2.xls:

Table S2. Full list of results from the Connectivity Map. Specificity score gives an estimate of how unique the similarities of the drugs' expression profile to the query expression profile are. A high score may indicate similarity is commonly seen in many drugs. Mean denotes the mean Connectivity score, a value between 1 and -1 that represents the strength and direction of similarity of a given drug signature to the query signature, positive scores indicate similarity, whereas negative scores indicate an opposite change in expression. Enrichment indicates the enrichment score, this value is calculated by subjecting the Connectivity score to a Kolmogorov-Smirnov statistical test, this value can then be used to produce probability values. For more details please see: https://www.broadinstitute.org/cmap/

SuppInfo 3.xlsx:

Table S4. Full list of differentially expressed genes.

SuppInfo 4.xlsx:

Table S5. Functional enrichment for genes differentially expressed under various treatments.

SuppInfo 5.xlsx:

Table S6. List of differentially expressed longevity-associated genes.

SuppInfo 6.xlsx:

Tabulated lifespan data for Figures 1, 2, S1, S2 and S3.

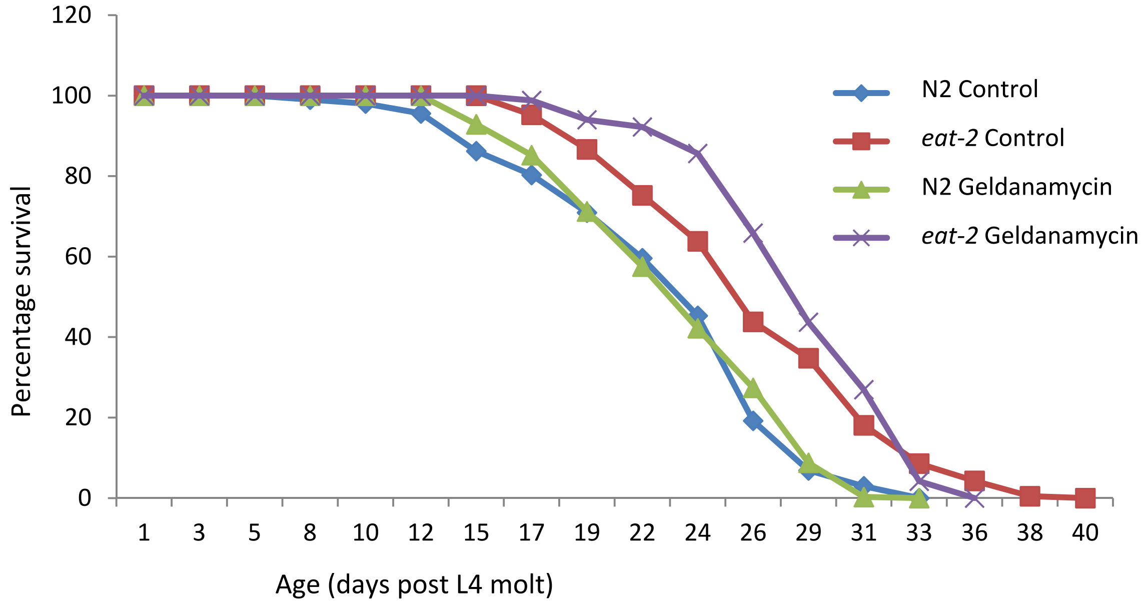

Figure S1. Percentage survival of wild-type (N2) or *eat-2* mutant worms alone or treated with geldanamycin. Worms unresponsive to touch stimulation were recorded as dead. Treated worms did not show any significant difference from controls of the same genotype.

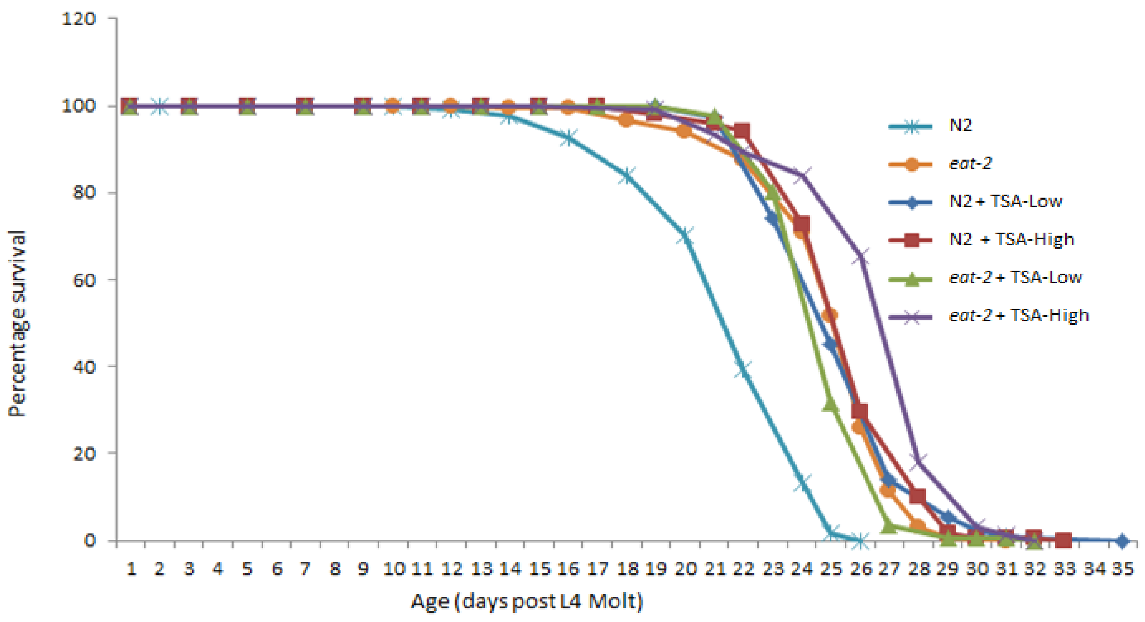

Figure S2. Percentage survival of wild-type (N2) or *eat-2* mutant worms alone or treated with a regular or double dose of trichostatin A (TSA). N2 worms treated with either 100µM or 200µM of TSA lived significantly longer than N2 (22.6% and 23.8%) and *eat-2* worms (6% and 7%). Treatment of *eat-2* worms with either dose led to a statistically significant increase in lifespan, however this was only a small increase (4.2% and 3.6%).

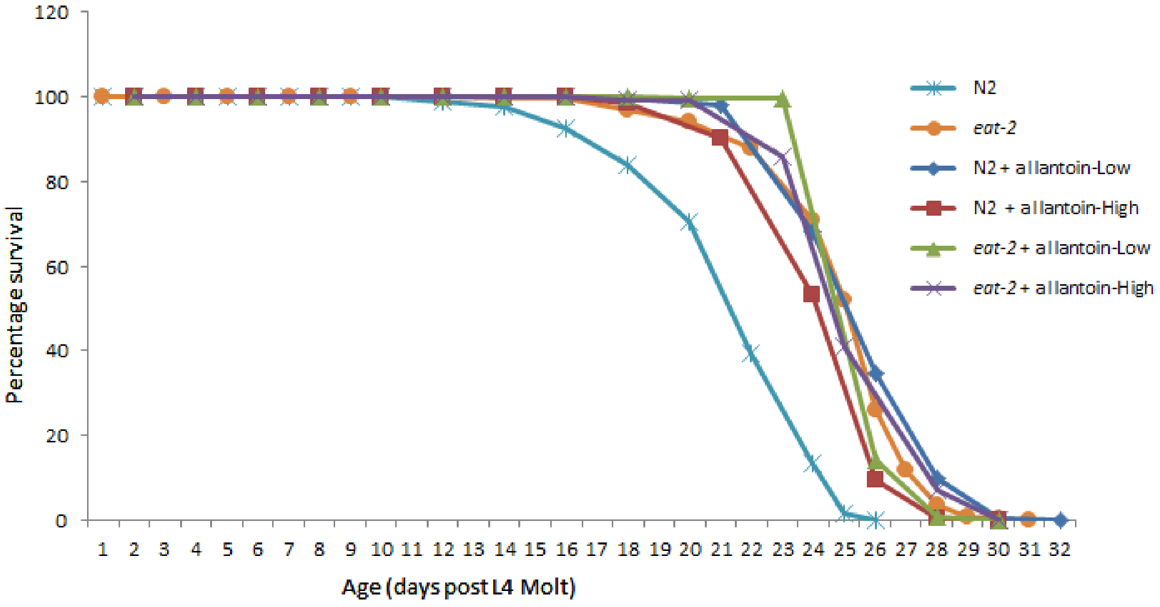

Figure S3. Percentage survival of wild-type (N2) or *eat-2* mutant worms alone or treated with a regular or double dose of allantoin. N2 worms treated with either 250µM or 500µM of allantoin lived significantly longer than N2 (20.1% and 14.3%). N2 worms treated with 250µM of allantoin but not 500µM was significantly longer lived than *eat-2* worms (3.9%). Treatment of *eat-2* worms with either dose led to a statistically significant increase in lifespan, however this was only a small increase (4.2% and 3.3%).

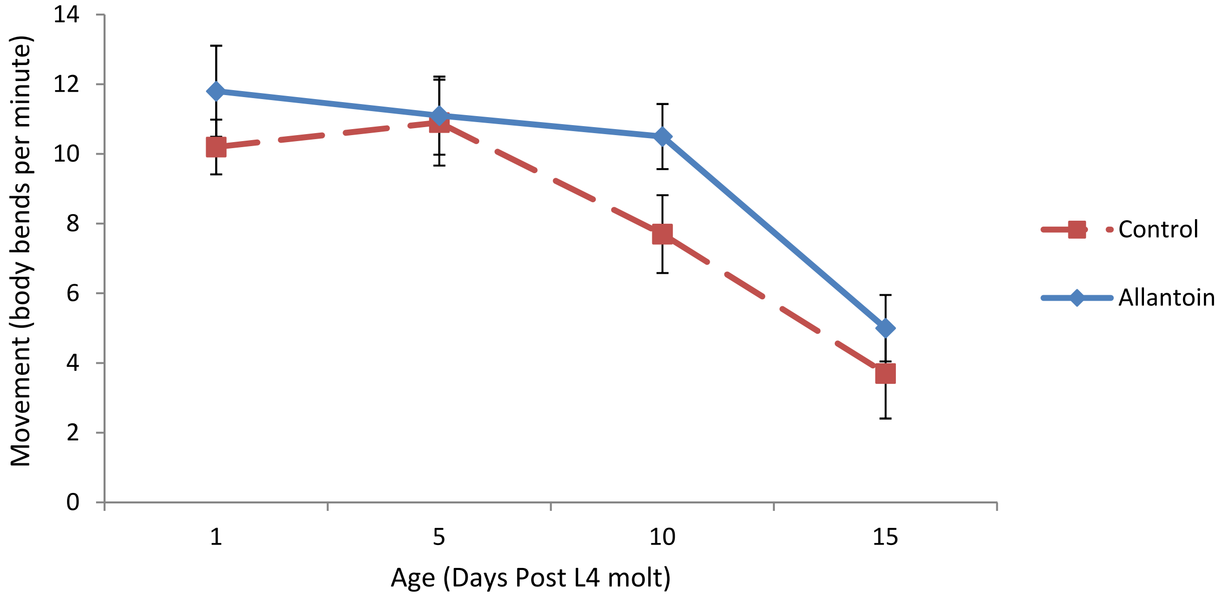

Figure S4. The movement rate measured as the number of body bends per minute of either N2 control worms or those treated with allantoin, trial 1. Movement rate was recorded on day 1, 5, 10 and 15 post L4 molt when drug treatment began. Allantoin treated worms showed a decline in movement that was not statistically different to controls. Error bars indicate +/- 1 standard error.

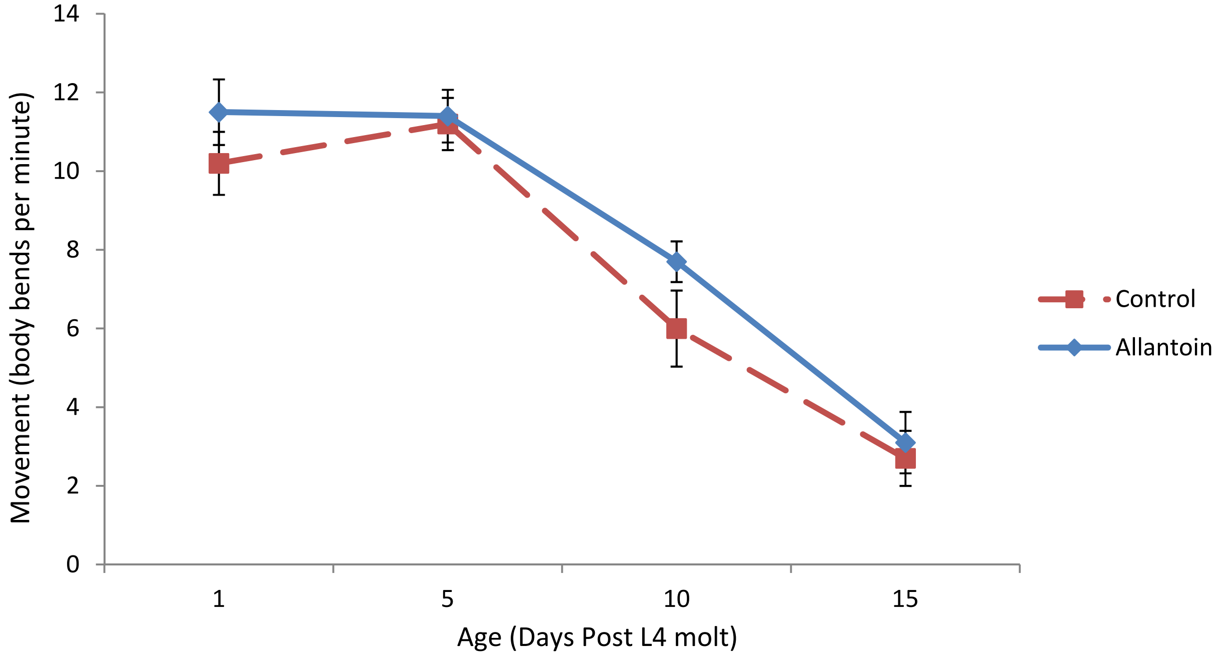

Figure S5. The movement rate measured as the number of body bends per minute of either N2 control worms or those treated with allantoin, trial 2. Movement rate was recorded on day 1, 5, 10 and 15 post L4 molt when drug treatment began. Allantoin treated worms showed a decline in movement that was not statistically different to controls. Error bars indicate +/- 1 standard error.

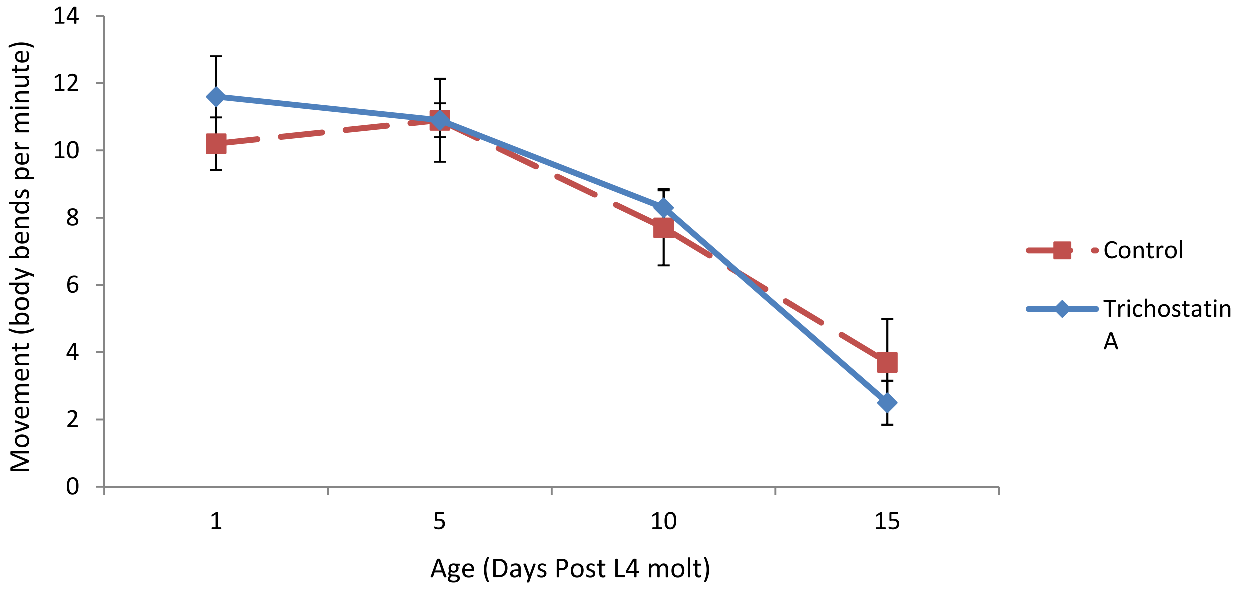

Figure S6. The movement rate measured as the number of body bends per minute of either N2 control worms or those treated with trichostatin A, trial 1. Movement rate was recorded on day 1, 5, 10 and 15 post L4 molt when drug treatment began. Trichostatin A treated worms showed a decline in movement that was not statistically different to controls. Error bars indicate +/- 1 standard error.

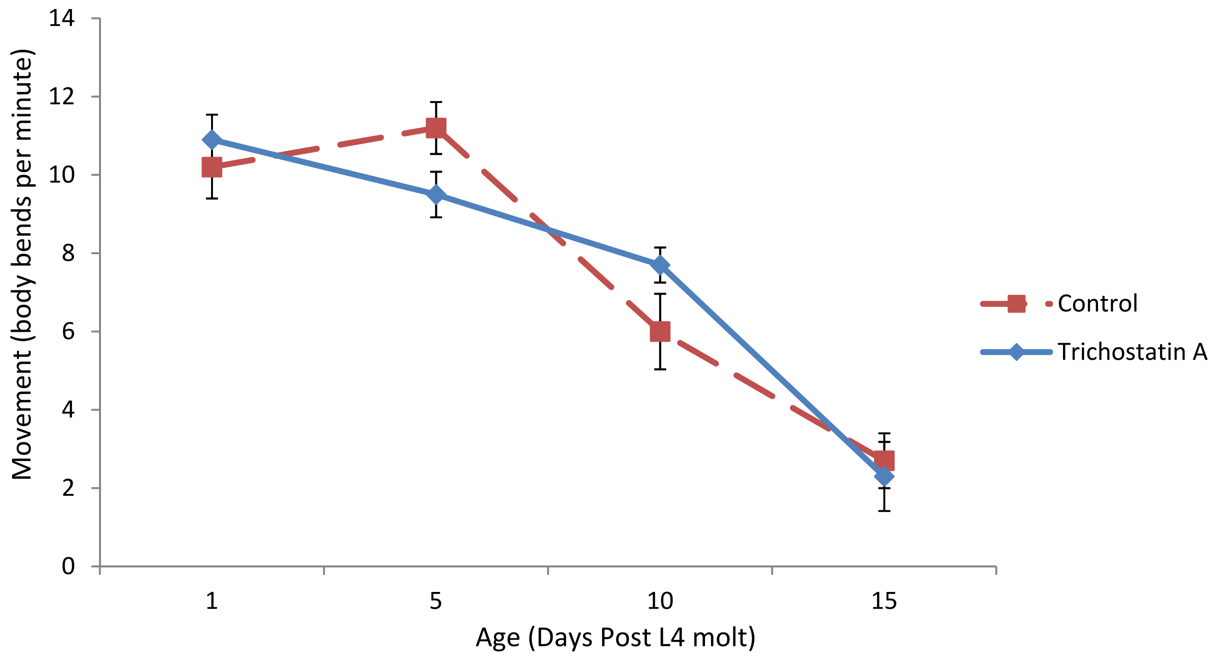

Figure S7. The movement rate measured as the number of body bends per minute of either N2 control worms or those treated with trichostatin A, trial 2. Movement rate was recorded on day 1, 5, 10 and 15 post L4 molt when drug treatment began. Trichostatin A treated worms showed a decline in movement that was not statistically different to controls. Error bars indicate +/- 1 standard error.

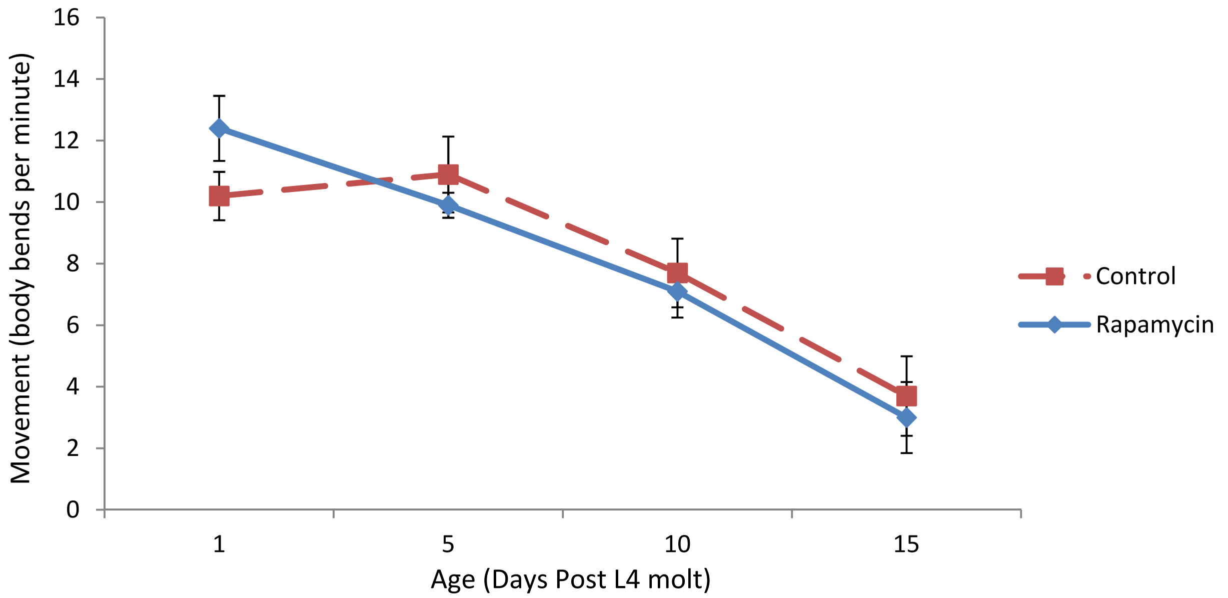

Figure S8. The movement rate measured as the number of body bends per minute of either N2 control worms or those treated with rapamycin, trial 1. Movement rate was recorded on day 1, 5, 10 and 15 post L4 molt when drug treatment began. Rapamycin treated worms showed a decline in movement that was not statistically different to controls. Error bars indicate +/- 1 standard error.

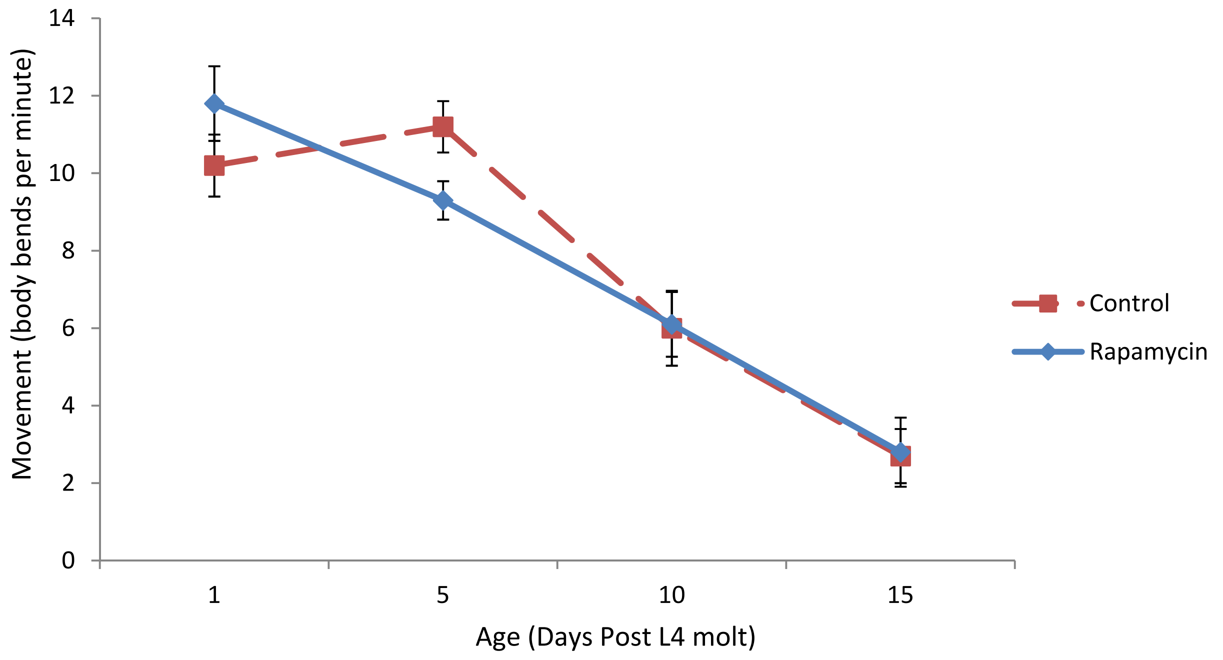

Figure S9. The movement rate measured as the number of body bends per minute of either N2 control worms or those treated with rapamycin, trial 2. Movement rate was recorded on day 1, 5, 10 and 15 post L4 molt when drug treatment began. Rapamycin treated worms showed a decline in movement that was not statistically different to controls. Error bars indicate +/- 1 standard error.

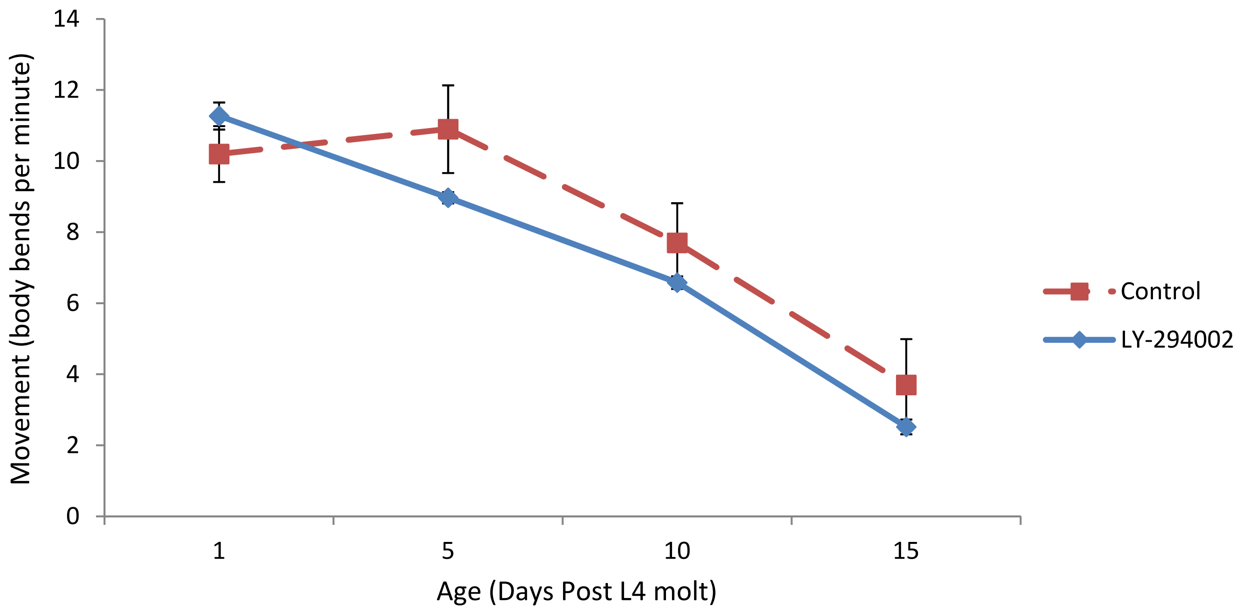

Figure S10. The movement rate measured as the number of body bends per minute of either N2 control worms or those treated with LY-294002, trial 1. Movement rate was recorded on day 1, 5, 10 and 15 post L4 molt when drug treatment began. LY-294002 treated worms showed a decline in movement that was not statistically different to controls. Error bars indicate +/- 1 standard error.

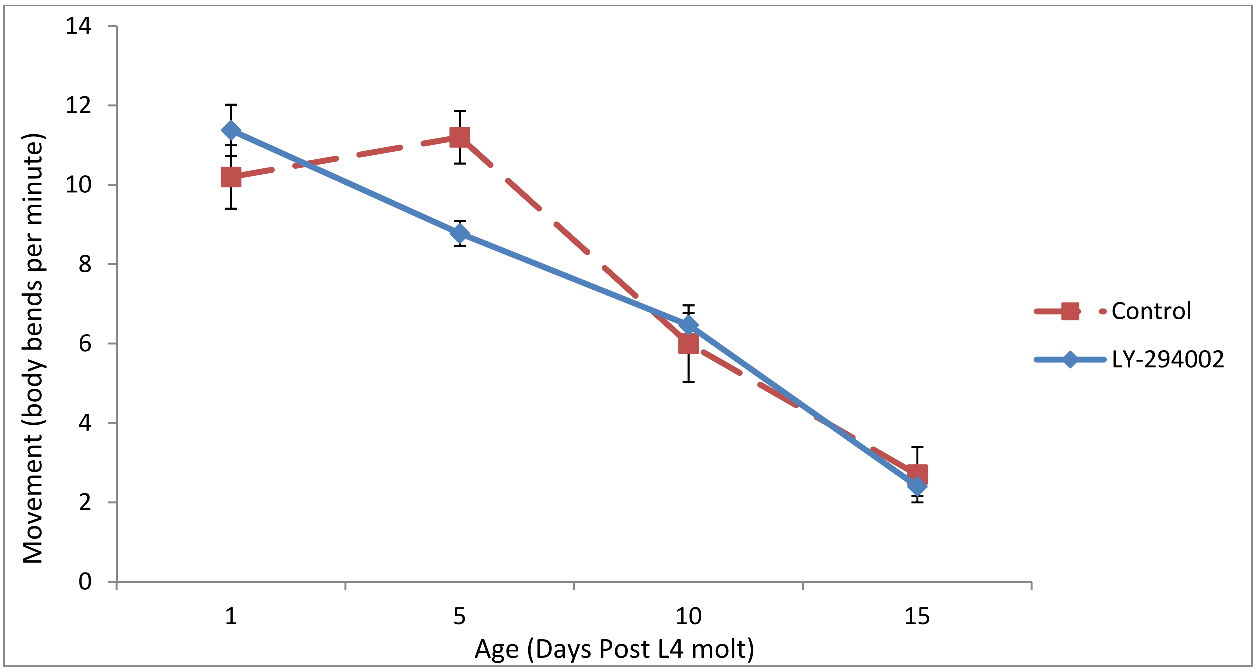

Figure S11. The movement rate measured as the number of body bends per minute of either N2 control worms or those treated with LY-294002, trial 2. Movement rate was recorded on day 1, 5, 10 and 15 post L4 molt when drug treatment began. LY-294002 treated worms showed a decline in movement that was not statistically different to controls. Error bars indicate +/- 1 standard error.

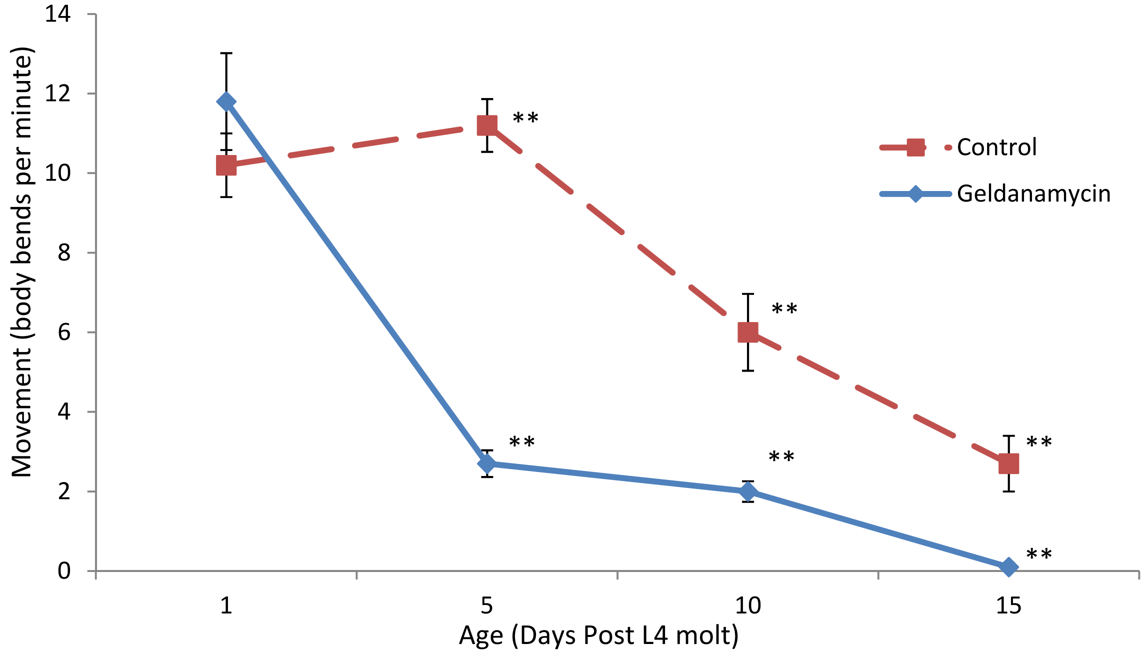

Figure S12. The movement rate of either N2 control worms or those treated with geldanamycin, trial 2. Movement rate was recorded on day 1, 5, 10 and 15 post L4 molt when drug treatment began. Geldanamycin treated worms show a much faster initial decline in movement compared to the controls. Movement rate decreases faster in geldanamycin treated worms with the rate of movement at day 5, 10 and 15 for treated worms (2.7, 2, 0.1 body bends per minute respectively) being lower than the rate in controls (11.2, 6, 2.7 body bends per minute respectively), to a significant degree two tailed T tests day 5: (P<0.001, tstat: 11.4383 df: 18 sd: 1.6617 N=10) day 10: (P<0.005, tstat: 4 df: 18 sd: 2.2361) day 15: (P<0.005 tstat: 3.6770 df: 18 sd: 1.5811 N=10). Stars (**) indicate a significance of at least 0.005. Error bars indicate +/- 1 standard error.

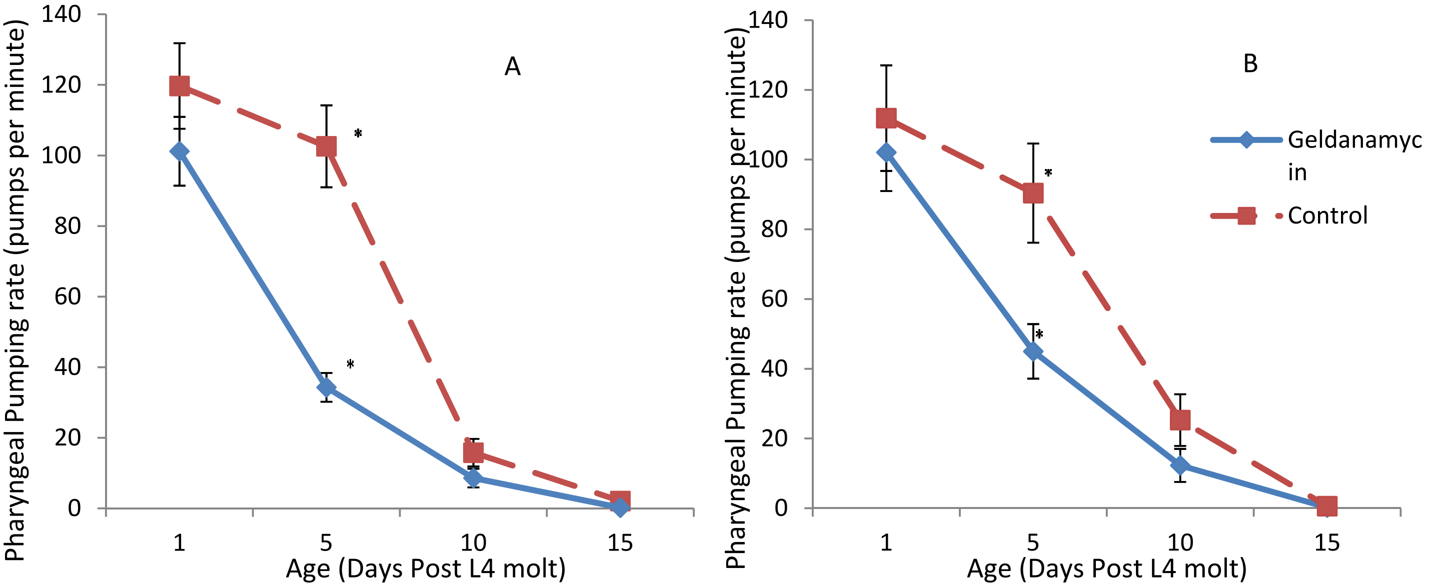

Figure S13. The Pharyngeal Pumping rate of either N2 control worms or those treated with geldanamycin. Pumping rate was recorded on day 1, 5, 10 and 15 post L4 molt when drug treatment began. Pumping rate in treated worms (45 pumps per minute) is lower than that of worms (90.4 pumps per minute) to a statistically significant degree at day 5 two tailed T test (P<0.05, tstat: 2.7957 df: 18 sd: 36.3122 N=10). A and B refer to two separate trials. Stars (*) indicate a significance of at least 0.05. Error bars indicate +/- 1 standard error.

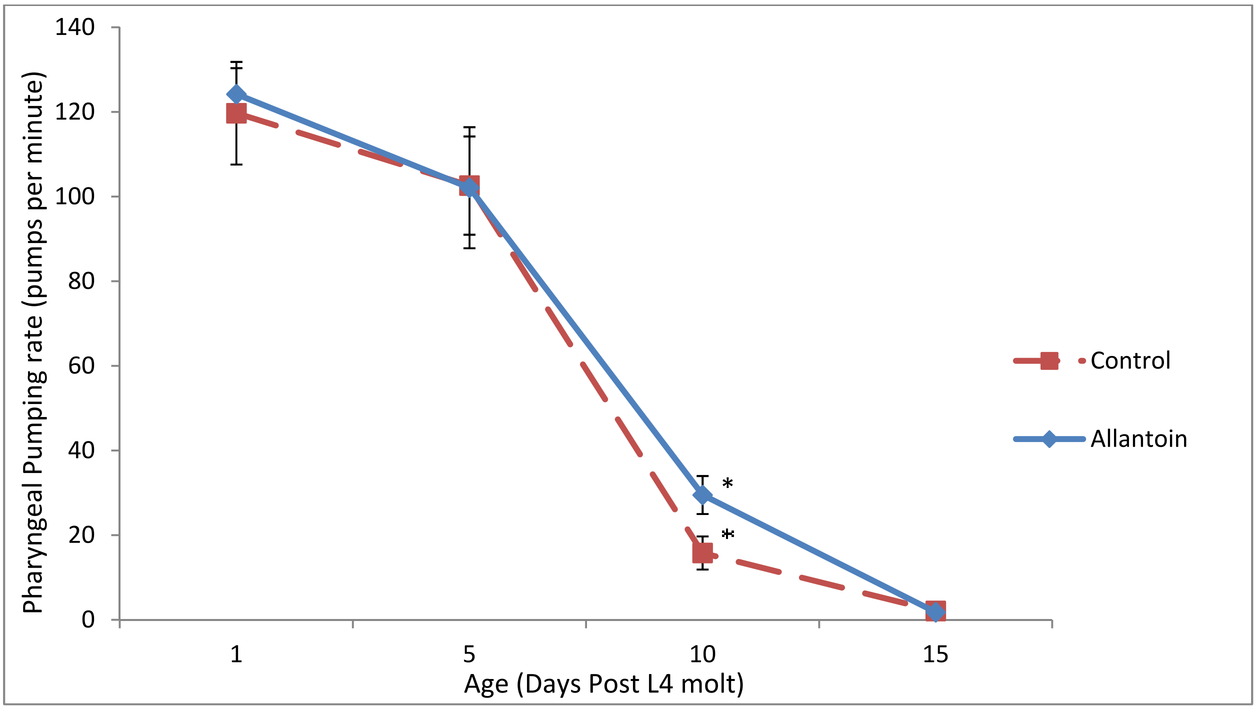

Figure S14. The Pharyngeal Pumping rate of either N2 control worms or those treated with allantoin, trial 2. Pumping rate was recorded on day 1, 5, 10 and 15 post L4 molt when drug treatment began. Pumping rate in treated worms (29.5 pumps per minute) is greater than that of controls (15.8 pumps per minute) to a statistically significant degree at day 10 (two tailed T test P<0.05, tstat: -2.3033 df: 18 sd: 13.3002 N=10). Stars (*) indicate a significance of at least 0.05. Error bars indicate +/- 1 standard error.

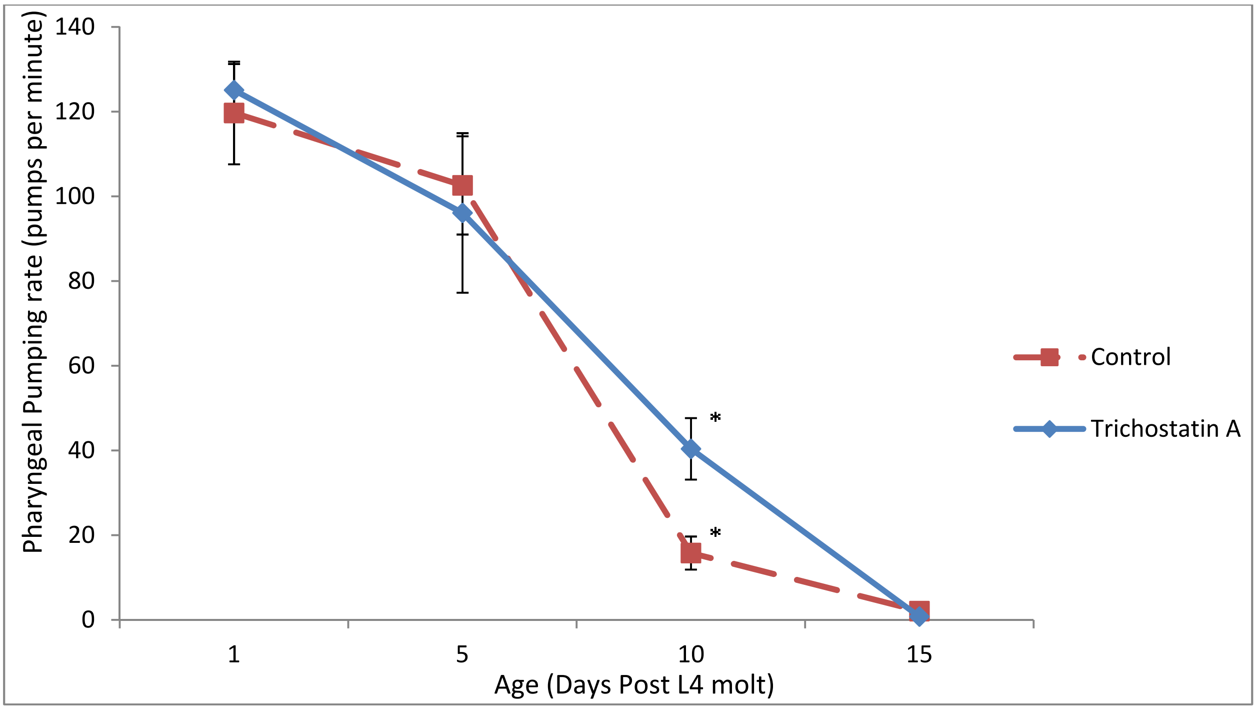

Figure S15. The Pharyngeal Pumping rate of either N2 control worms or those treated with trichostatin A, trial 2. Pumping rate was recorded on day 1, 5, 10 and 15 post L4 molt when drug treatment began. Pumping rate in treated worms (40.4 pumps per minute) is greater than that of controls (15.8 pumps per minute) to a statistically significant degree at day 10, two tailed T test (P<0.01, tstat: -2.9861 df: 18 sd: 18.4210 N10). Stars (*) indicate a significance of at least 0.05. Error bars indicate +/- 1 standard error.

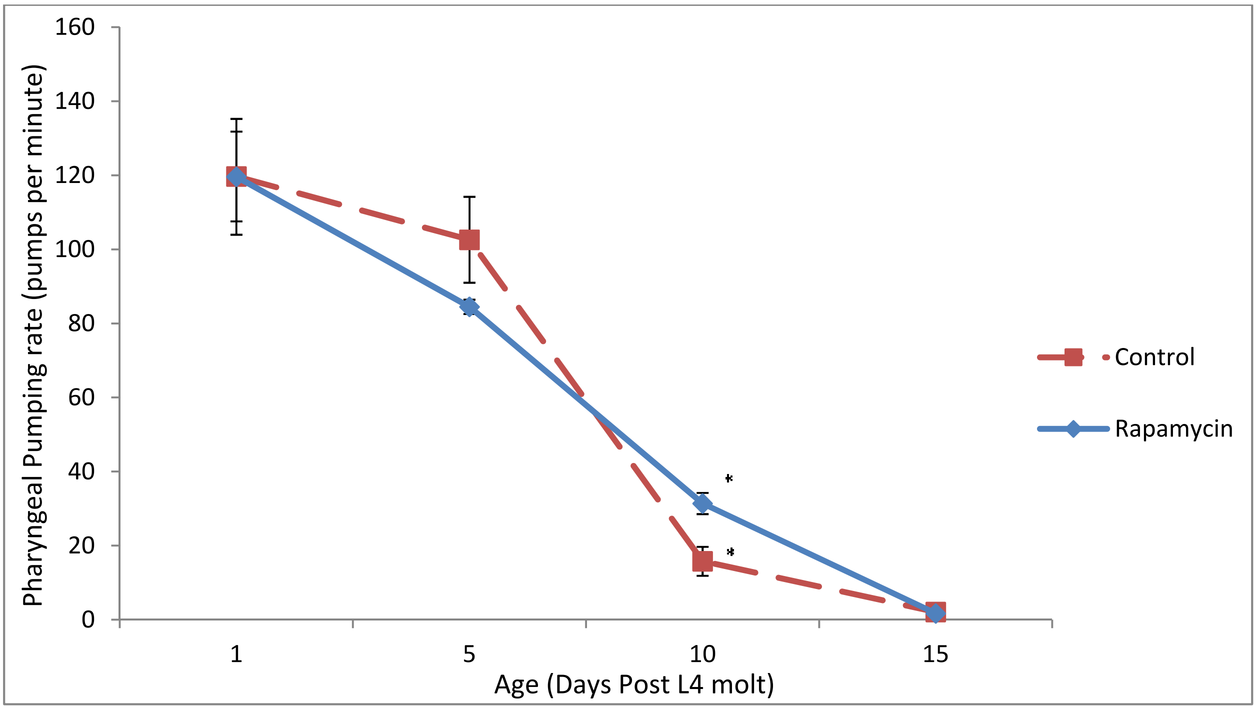

Figure S16. The Pharyngeal Pumping rate of either N2 control worms or those treated with Rapamycin, trial 2. Pumping rate was recorded on day 1, 5, 10 and 15 post L4 molt when drug treatment began. . Pumping rate of treated worms (31.4 pumps per minute) was greater than that of controls (15.8 pumps per minute) to a statistically significant degree at day 10 two tailed T test (P<0.005, tstat: -3.2203 df: 18 sd: 10.8321 N=10). Stars (*) indicate a significance of at least 0.05. Error bars indicate +/- 1 standard error.

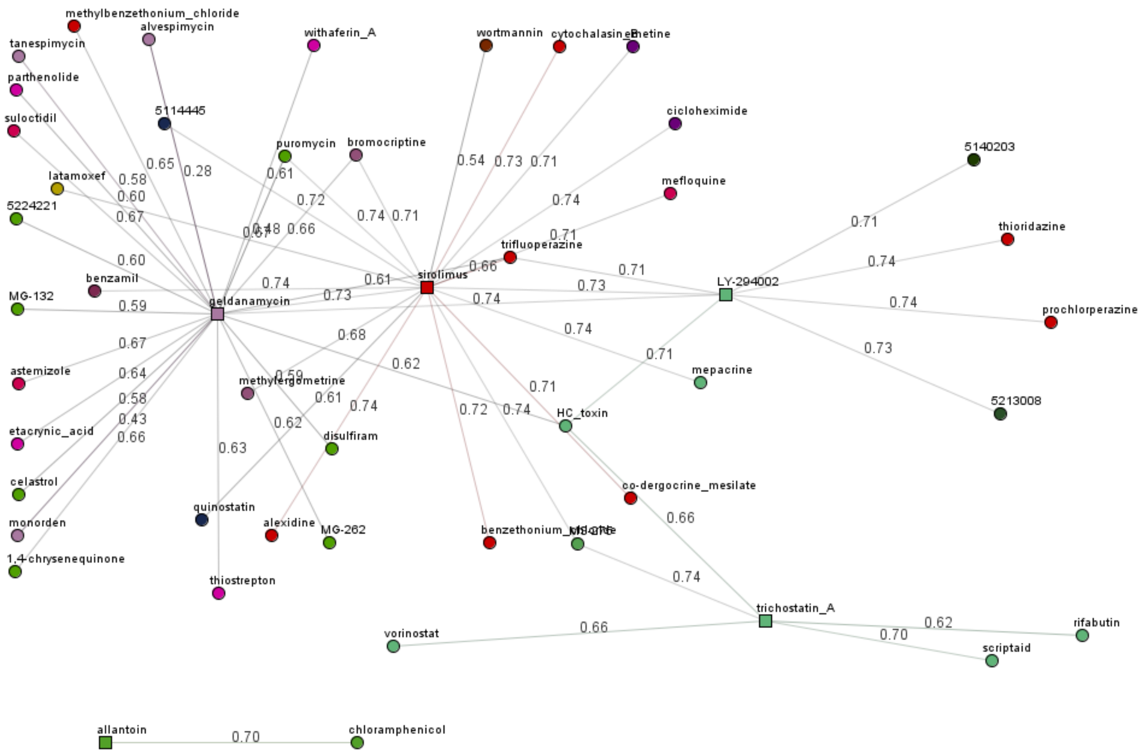

Figure S17. Mantra 2.0 analysis of the compounds under study. Each square represents one of the drugs under investigation. Each numbered line represents the similarity of induced gene expression profile of each compound. Geldanamycin, rapamycin (sirolimus), and LY-294002 seem to be relatively similar in their induced gene expression profiles. Trichostatin A seems to be more distinct with only a indirect connection to these drugs. The analysis used a 0.75 cut off as larger cut offs produced too many nodes to discern any useful information.

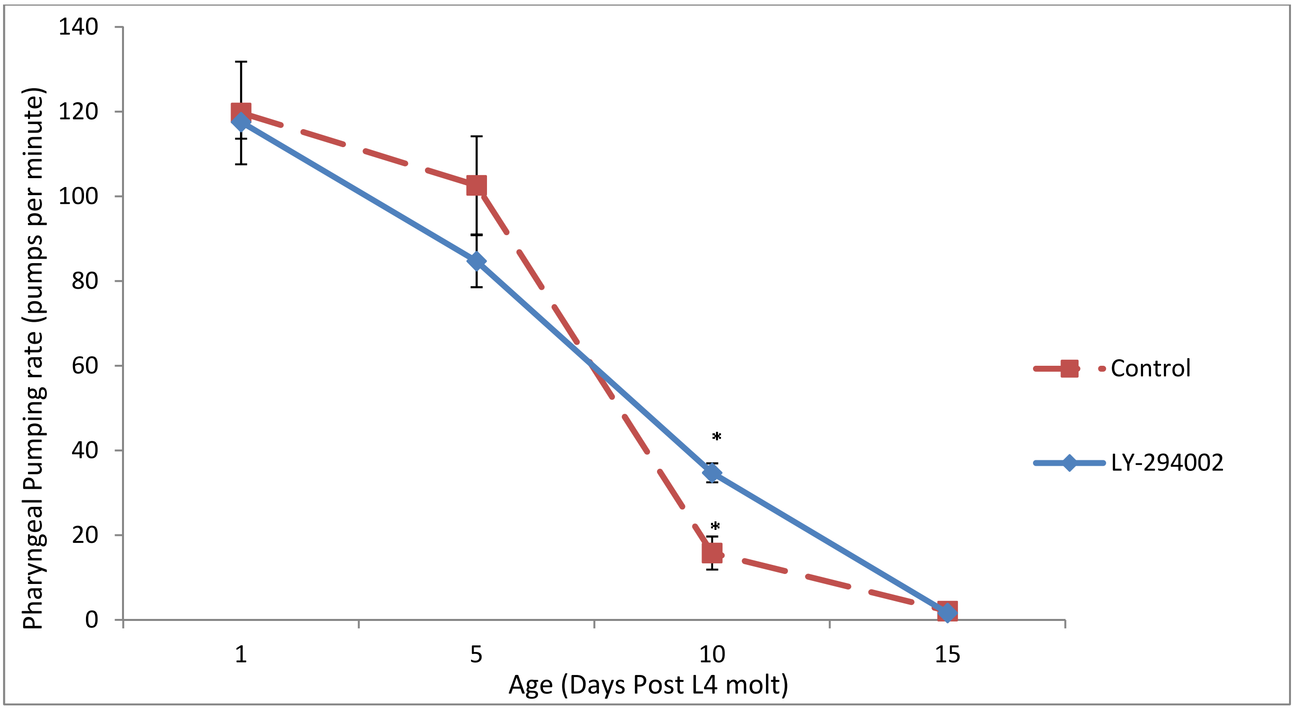

Figure S18. The Pharyngeal Pumping rate of either N2 control worms or those treated with LY-294002, trial 2. Pumping rate was recorded on day 1, 5, 10 and 15 post L4 molt when drug treatment began. Pumping rate of treated worms (34.7 pumps per minute) was greater than that of controls (15.8 pumps per minute) to a statistically significant degree two tailed T test ($P<0.001$, tstat: -5.7496 df: 18 sd: 18.1230 N=10). Stars (*) indicate a significance of at least 0.005. Error bars indicate +/- 1 standard error.

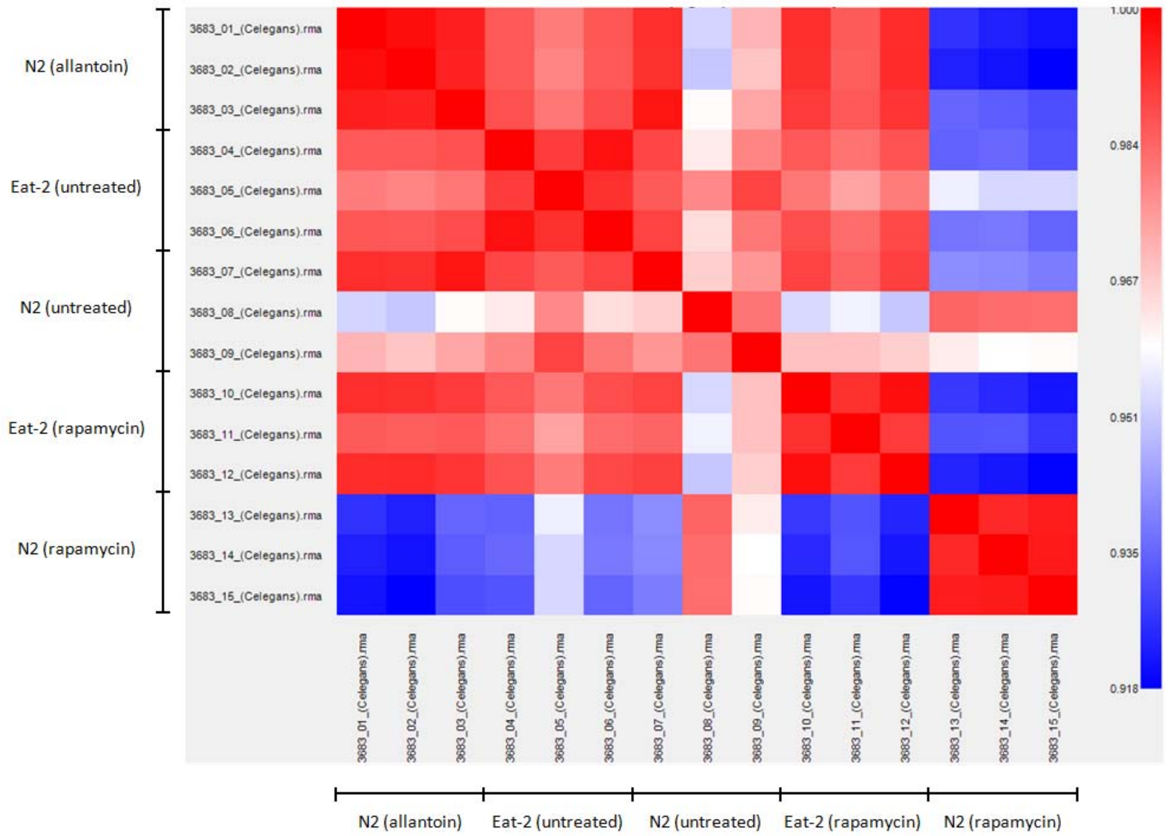

Figure S19. Pearson's correlation of the signal (using RMA-normalized gene-level signals) between samples, obtained from the Affymetrix Expression Console.
