# Supplementary material for: A network pharmacology approach reveals new candidate caloric restriction mimetics in C. elegans
Source: Aging Cell. 2015 Dec 16;15(2):256–66. doi: 10.1111/acel.12432 (PMC4783339; doi:10.1111/acel.12432)
Supplement: Supplementary file 2 — Table S1 Human genes used to query the Connectivity Map and their relative change in the CR transcriptional profile used. Table S3 The variation seen in lifespan compared to controls of each treatment. [file ACEL-15-256-s007.pdf]

Table S1. Human genes used to query the Connectivity Map and their relative change in the CR transcriptional profile used.

	Genes
Up	MDM2, HSD17B4, RPS8, HSPA8, SMPD2, KPNB1, DNAJB6 /// TMEM135, CXXC1, SETDB1, CA4, JAK1, ING1, WRNIP1, KPNB1, POLR2A, DNAJB6, CA4, MOGS, UBB, SUCLA2, KPNB1, UBB, ARNT, CRYZ, CCT7, RPS8 /// RPS8P8
Down	CAPRIN1, RPL23, RPL3, ACTG1, PPP3CB, MAP7, FDFT1, TFPI, RBBP4, LOC100294182 /// RPL3, LAMA1

Table S3. The variation seen in lifespan effects of drugs compared to controls in N2 and *eat-2* worm strains. The maximum and minimum change in lifespan in all of the trials performed and the number of trials performed of each lifespan assay in drug-treated *C. elegans*. Abbreviations: TSA

Treatment	% Difference observed	Number of replicates
N2 + RAPAMYCIN	10.4 to 22.8	5
N2+ TSA	8.71 to 23.2	5
N2 + GELDANAMYCIN	1.52 to 3.06	4
N2 + LY-294002	13.8 to 22.8	4
N2 + ALLANTOIN	20.4 to 25.0	3
eat-2 + RAPAMYCIN	-3.30 to 10.7	5
eat-2 + TSA	-0.317 to 5.81	5
eat-2 + GELDANAMYCIN	-20.9 to 9.71	4
eat-2 + LY-294002	-1.15 to 13.6	4
eat-2 + ALLANTOIN	1.97 to 8.63	3
